# Characterization and genomic analysis of a lytic *Stenotrophomonas maltophilia* short-tailed phage A1432 revealed a new genus of the family *Mesyanzhinovviridae*

**DOI:** 10.3389/fmicb.2024.1400700

**Published:** 2024-06-27

**Authors:** Shixia Li, Man Xu, Deying Yang, Mei Yang, Hejing Wu, Xuelian Li, Changzhou Yang, Zheng Fang, Qingshan Wu, Leitao Tan, Wei Xiao, Qingbei Weng

**Affiliations:** ^1^School of Life Sciences, Guizhou Normal University, Guiyang, China; ^2^Yunnan Institute of Microbiology, Yunnan International Joint Laboratory of Virology and Immunology, Yunnan University, Kunming, China; ^3^Qiannan Normal University for Nationalities, Duyun, China

**Keywords:** *Stenotrophomonas maltophilia*, phage, *Mesyanzhinovviridae*, *Bradleyvirinae*, karst cave, new genus, lytic phage

## Abstract

*Stenotrophomonas maltophilia* (*S. maltophilia*) is an emerging opportunistic pathogen that exhibits resistant to a majority of commonly used antibiotics. Phages have the potential to serve as an alternative treatment for *S. maltophilia* infections. In this study, a lytic phage, A1432, infecting *S. maltophilia* YCR3A-1, was isolated and characterized from a karst cave. Transmission electron microscopy revealed that phage A1432 possesses an icosahedral head and a shorter tail. Phage A1432 demonstrated a narrow host range, with an optimal multiplicity of infection of 0.1. The one-step growth curve indicated a latent time of 10 min, a lysis period of 90 min, a burst size of 43.2 plaque-forming units per cell. *In vitro* bacteriolytic activity test showed that phage A1432 was capable to inhibit the growth of *S. maltophilia* YCR3A-1 in an MOI-dependent manner after 2 h of co-culture. BLASTn analysis showed that phage A1432 genome shares the highest similarity (81.46%) with *Xanthomonas* phage Xoo-sp2 in the NCBI database, while the query coverage was only 37%. The phage contains double-stranded DNA with a genome length of 61,660 bp and a GC content of 61.92%. It is predicted to have 79 open reading frames and one tRNA, with no virulence or antibiotic resistance genes. Phylogenetic analysis using terminase large subunit and DNA polymerase indicated that phage A1432 clustered with members of the *Bradleyvirinae* subfamily but diverged into a distinct branch. Further phylogenetic comparison analysis using Average Nucleotide Identity, proteomic phylogenetic analysis, genomic network analysis confirmed that phage A1432 belongs to a novel genus within the *Bradleyvirinae* subfamily, *Mesyanzhinovviridae* family. Additionally, phylogenetic analysis of the so far isolated *S. maltophilia* phages revealed significant genetic diversity among these phages. The results of this research will contribute valuable information for further studies on their morphological and genetic diversity, will aid in elucidating the evolutionary mechanisms that give rise to them.

## 1 Introduction

*Stenotrophomonas maltophilia* is a non-fermentating Gram-negative bacteria that ubiquitously distribute in the earth’s biosphere (Kalidasan et al., 2018). It is a significant opportunistic pathogen, causing a variety of infections including pneumonia, meningitis endocarditis, catheter-associated bacteremia or septicemia prevalent in both hospital and community infections. *S. maltophilia* has been one of the most frequent causes of respiratory infections, particularly in intensive care unit (ICU), surpassing *Acinetobacter baumannii*, *Pseudomonas aeruginosa*, *Klebsiella pneumoniae* in prevalence (Zhang et al., 2021; Kullar et al., 2022). Due to the induction of chromosomally encoded *β*-lactamase, multidrug efflux pump, a less permeable outer membrane, *S. maltophilia* has intrinsic resistance to most antibiotics, including *β*-lactam antibiotics, cephalosporins, carbapenems (McCutcheon and Dennis, 2021), which limits treatment options for *S. maltophilia* infections. Therefore, alternative treatments for bacterial infections, especially for infections caused by multidrug-resistant (MDR) bacteria, are particularly important.

Bacteriophages, or phages, are natural enemies of bacteria that are able to specifically infect and kill their host bacteria. Lytic phages present against most bacteria by overcoming their defense systems (Rimon et al., 2023). With the rise in bacterial drug resistance over recent decades, phages are considered to be a promising alternative to antibiotics for controlling drug-resistant pathogens. Phages offer several advantages over antibiotics or antibacterial agents, such as their specificity in lysing host bacteria, their ability of self-dosing by lysing host bacteria to replicate, their capacity to kill their host without disrupting the normal microbial flora, thereby preventing bacterial disorder and secondary infections (Yeh, 2017). Therefore, phage therapy, which utilizes obligatory lytic phages to kill their host, is regaining attention as a potential treatment option for bacterial infections (Assefa, 2022). This approach also offers new possibilities for preventing and treating infections caused by drug-resistant *S. maltophilia*.

Microbial resources form the foundation of microbial product development. Previous analysis of 89 published whole-genome sequences of *S. maltaphilia* has revealed a high degree of genetic diversity among intra-species strains of *S. maltaphilia*. This analysis also predicted a high prevalence and genetic diversity of prophages, as well as a significant number of uncharacterized phages within the *S. maltaphilia* genome (Fang et al., 2023). To date, only 68 *S. maltophilia* phages have been sequenced,^1^ with just 44 of these having been characterized. These phages, primarily isolated from hospitals, wastewater, soil, have mostly been described as tailed phages belonging to the family *Mesyanzhinovviridae*, *Autographiviridae* and *Schitoviridae*. It has been reported that *S. maltophilia* phages can be used as potential agents to improve the survival rate of mice infected with *S. maltophilia* (Han et al., 2022a; Han P. et al., 2022), can eliminate the biofilm produced by *S. maltophilia* (Fanaei Pirlar et al., 2022). Although a number of virulent *S. maltophilic* phages have been isolated, given the diversity and intrinsic resistance to antibiotic of *S. maltophilia*, more phages should be isolated for phage therapy.

Recently, the International Committee on Taxonomy of Viruses (ICTV) adopted a roadmap for classifying phages based on their genomes (Turner et al., 2021). The families *Podoviridae*, *Siphoviridae*, *Myoviridae*, the order *Caudovirales* were abolished, numerous new floating subfamilies and genera emerged (Turner et al., 2023). Therefore, efforts to isolate phages are critical for the correct classification and understanding of the evolutionary relationships of viruses. There is an urgent need to expand phage libraries and further our understanding and resources in this field.

Karst caves are considered as extreme environments characterized by poor nutrition, darkness, oxygen deprivation. Despite the total organic carbon content in these caves being less than 2 mg/L, they are rich in microbial resources (Dong et al., 2020) with the average number of microbes in cave rock reaching as high as 10^6^ g^−1^ (Tomczyk-Żak and Zielenkiewicz, 2016). Phages, the most abundant entities in the biosphere, remain largely unexplored within these caves. In this study, a novel lytic phage A1432 was isolated from cave sediments using *S. maltophilia* YCR3A-1, a strain also isolated from karst caves, as the host. The biological and genomic characteristics of phage A1432 were characterized, providing evidence that this phage represents a novel genus within the family *Mesyanzhinovviridae*. These findings not only offer new resources for *S. maltophilia* phage therapy but also contribute valuable insights on the morphological and genetic diversity of phages, aiding in the elucidating of their evolutionary mechanisms.

## 2 Materials and methods

### 2.1 Sampling

Sediment samples were collected from a karst cave (107°47′29”N, 25°26′12″E) in Libo County, Guizhou Province, China, in July 2019. The average temperature was 16.8°C, the average CO_2_ concentration was 480.58 PPM in the cave. Samples were obtained from the rock surface at intervals of every 10 meters from three different sites within the cave. Subsequently, these samples were then pooled, transferred into sterile tubes, chilled on ice and transported back to 4°C for storage for further bacterial and phage isolation in the laboratory.

### 2.2 Bacterial strains and culture, antimicrobial susceptibility testing

Bacterial strain was isolated from sediment samples using the spread plate method with Luria-Bertani (LB) media and purified 3–5 times to obtain a strain with a consistent colony morphology, designated as YCR3A-1. Sterile glycerol was added to the strain at a final concentration of 25% and stored at −80°C for subsequent experiments. The 16S rRNA gene of YCR3A-1 was sequenced by Sangon Biotech (Shanghai) Co., Ltd., the sequencing data was submitted to the National Center for Biotechnology Information (NCBI).

Ten *S. maltophilia* strains (originating from clinical samples) were kindly provided by Professor Huahao Fan, College of Life Science and Technology, Beijing University of Chemical Technology (Han et al., 2021), five *Stenotrophomonas* spp. were isolated from caves in our laboratory. Totally 15 *Stenotrophomonas* spp. were used to test the host range of phage A1432 (Supplementary Table S1). Based on 16S rRNA gene sequence, a phylogenetic tree of YCR3A-1 with similar strains and the 15 strains of *Stenotrophomonas* spp. was constructed using the maximum likelihood (ML) method in MEGA software with 1,000 bootstrap replicates (Kumar et al., 2016).

All strains were cultured in LB broth or on LB agar plates at 37°C. AST was performed using the disk diffusion method as previously described with some modifications (Han et al., 2022a). Briefly, bacteria were incubated to the exponential phase (OD600 = 0.7) at 37°C, 1 mL of the bacterial culture was added to 10 mL of 0.7% agar-containing LB medium. This mixture was immediately poured onto Petri dishes. After solidification, antibiotic sensitivity disks (Hunan BKMAM Biotechnology Co., Ltd.) were placed on the plates and incubated overnight at 37°C. The antibiotics used are listed in Supplementary Table S1. Sterile antibiotic-free paper disks were used as blank controls, *E. coli* DH5α was used as a negative control under the same experimental conditions. If a strain is sensitive to a particular antibiotic, a clear zone of inhibition forms around the disk. The diameter of the zone is measured and recorded. The experiment was conducted three times.

### 2.3 Isolation, purification and propagation of phage

Phage A1432 was isolated from a sediment sample from a karst cave, using the host strain YCR3A-1. Initially, 10 g of the collected sediments were homogenized in 90 mL sodium chloride-magnesium sulfate (SM) buffer (100 mM NaCl, 8 mM MgSO_4·_7H_2_O, 50 mM Tris–HCl [pH 7.5]). The resulting supernatant was filtered through a 0.22 μm poresize membrane (Millipore). The collected filtrate was then subjected to the double-agar overlay method to detect the presence of phages. Briefly, a series of dilutions of the filtrate containing the potential phages was prepared. A total of 1 mL of the host YCR3A-1 in the logarithmic growth phase in LB broth was added to 1 mL of appropriately diluted filtrate. After a 20 min incubation at 37°C, the mixture was poured onto solid LB agar, 8 mL of semi-solid medium (LB broth with 0.5% agar) melted at 50°C was added. Once dried, the dishes were kept upside-down and incubated overnight at 37°C. The appearance of plaques indicated a successful infection of the host bacterium by the phages.

In order to purify the isolated phages, a clear single plaque was individually transferred into SM buffer to obtain the first purified phage. This purification process was repeated three times for each phage, the fully purified phage was then mixed with a fresh culture of the host strain and incubated at 37°C with shaking at 180 rpm to obtain the phage proliferation solution. To remove cell debris, the proliferation solution was centrifuged at 8000 g for 30 min at 4°C and then aseptically filtered through a 0.22 μm filter membrane. The phage titer in the filtrate was determined by the double-layer agar method and expressed as plaque-forming units (PFU) per milliliter of filtrate. The proliferating solution was stored at 4°C for subsequent experiments. Sterile glycerol at a final concentration of 25% was added to the phage proliferating solution and stored at −80°C for long periods of time.

### 2.4 Transmission electron microscopy

Phage supernatant was centrifugated at 12,000 g for 2 min and then filtered through a 0.22 μm membrane. A droplet of phage suspension (10^9^ PFU/ml) was applied onto a carbon-coated copper grid for 10 min. The grids were negatively stained with 2% (w/v) phosphotungstic acid (pH 6.8) for 5 min, air-dried, then observed with an HT7800 transmission electron microscope (HITACHI, Tokyo, Japan) at an accelerating voltage of 100 kV (Yi et al., 2022).

### 2.5 Host range of phage

Fifteen strains of *Stenotrophomonas* genus were used to investigate the host range of phage A1432 (Supplementary Table S1). Five μL of the purified phage suspension (10^8^ PFU/mL) was inoculated onto the relevant bacterial culture plates and incubated at 37°C for 24 h to observe the formation of lysis zones at the phage suspension. All tests were performed in triplicate.

### 2.6 Multiplicity of infection and one step growth curve

The MOI represents the ratio of phages to host bacteria during infection. To determine the optimal MOI for A1432, YCR3A-1 culture was mixed with the phage in different proportions: 100, 10, 1, 0.1, 0.01 and 0.001. The mixtures were incubated for 15 min at 37°C, centrifuged, the pellet was resuspended in 2 mL of fresh media. The samples were incubated for 3 h at 37°C, after which phage titer was determined using the double-layer agar method with A1432 as the host. The MOI that resulted in the highest titer was identified as the optimal MOI. Each experiment was performed in triplicate.

The one-step growth curve was performed. Briefly, the phage was mixed with the host bacteria according to the optimal MOI. After adsorption for 15 min at 37°C, the mixture was centrifuged and the pellets were resuspended in 50 mL of LB broth, followed by incubation at 37°C under shaking at 180 rpm. Samples were taken at 0, 10, 20, 30, 40, 70, 100, 130, 160, 190, 220 and 250 min after inoculation. The phage titer was determined using double-layer agar method. The experiment was repeated three times.

### 2.7 Physiochemical stability of phage

In the thermal assay, phages (10^12^ PFU/mL) were incubated at 0, 4, 20, 30, 40, 50, 55, 60, 70 and 80°C for 60 min, respectively. In the pH assay, 100 μL of phage solution (10^12^ PFU/mL) was added to 900 μL of SM buffer with different pH values and then incubated at 37°C for 60 min. The pH value of the SM buffer was adjusted from 2 to 13 using HCl or NaOH (Assefa, 2022). The phage titer was measured using a double-layer agar technique. Each experiment was performed in triplicate.

The sensitivity of the phage to chloroform is often used as an important reference for determining the presence or absence of lipid components in phage capsids (Kalidasan et al., 2018). Phage A1432 (1012 PFU/mL) was mixed with chloroform at a final concentration of 25% and incubated for 10 min at room temperature (Xiang et al., 2020). The supernatant was collected after centrifugation at 13,000 g for 1 min, the phage titer was assayed by the double-layer agar method. SM buffer instead of chloroform was used as a blank control.

### 2.8 *In vitro* bacteriolytic activity

The bactericidal activity of phage A1432 was evaluated *in vitro* as previously described, with a few modifications (Chen et al., 2023). Briefly, 100 μL of phage A1432 was mixed with host YCR3A-1 (OD_600_ = 0.30) in the equal volume at MOI of 0.01, 0.1, 1, 10, respectively, incubated at 37°C for 24 h. Bacterial growth was monitored by measuring the OD600 at each time point using spectrophotometry. The experiment was conducted three times. A culture medium of host YCR3A-1 without phage was used as a positive control.

### 2.9 Sequencing of phage genome

The purified phage lysate (10^9^ PFU/ml) was filtered through 0.22 μm pore size filter (Millipore). Then DNase I and RNase A (Takara, Japan) were added to 200 μL phage lysate (10^9^ PFU/mL) at final concentrations of 50 U/mL and 250 μg/mL, respectively, the mixture was then treated for 1 h at 37°C to remove from DNA and RNA. After digestion, genomic DNA of phage A1432 was extracted using the TaKaRa MiniBEST viral RNA/DNA extraction kit (Takara, Japan) following the manufacturer’s instructions. DNA was finally resuspended in deionized water and stored at −20\u00B0C. The concentration and quality of DNA were determined at 260 nm and 280 nm using Nanodrop spectrophotometry.

DNA libraries were constructed using the TruSeqTM DNA Sample Prep Kit (Illumina, San Diego, CA, United States) and sequenced in paired-end model by Personalbio (Shanghai Personal Biotechnology Co., Ltd., China) through the Illumina NovaSeq platform. The obtained FastQ raw reads were trimmed of adaptors and low-quality bases and short reads were filtered using AdapterRemoval v2.1.3 (Schubert et al., 2016). *De novo* assembly was performed using A5-MiSeq v20160825 and SPAdes v3.12.0 software (Bankevich et al., 2012; Coil et al., 2014). Pilon v1.18^2^ was used to correct the results for obtaining the final genome sequence (Walker et al., 2014).

### 2.10 Genome analysis

After obtaining the whole genome sequence, open reading frames (ORFs) in the phage genome were identified using the RAST server^3^ (Aziz et al., 2008). The putative function of each ORF-encoded protein was annotated by searching against the non-redundant protein sequences (NR) database with BLASTp,^4^ tRNAscan-SE was used to search for tRNAs.^5^ The presence of determinants of antimicrobial resistance was investigated using the comprehensive Antibiotic Resistance Database^6^ (Alcock et al., 2023) and the Virulence Factor Predictor^7^ (Liu et al., 2019). The genome circle map of phage A1432 was drawn by CGView server (Grant and Stothard, 2008).

### 2.11 Phylogenetic analysis and comparative genomic analysis

The phage terminase large subunit (TerL) and DNA polymerase are highly conserved characteristic proteins that are frequently utilized in virus classification. The phylogenetic trees were constructed by Maximum Likelihood (ML) method using MEGA 7.0 software (Kumar et al., 2016) with the JTT matrix-based model and 1,000 bootstrap replicates based on the amino acid sequences of TerL and DNA polymerase I Moreover, a proteome phylogenetic tree of phage A1432 was generated using the Viral Proteomic Tree server^8^ (Nishimura et al., 2017). In these different analyses, BLASTp (TerL and DNA polymerase I) or BLASTn (proteomic tree) with an E-value cutoff of 0 (accessed 4 May 2024) were used to find the 20 best hit viruses in the GenBank database, duplicate and unassociated sequences were removed. Then, the same viral genome dataset containing the 20 best hit phages was finally used (only 19 viruses were selected to construct the TerL phylogenetic tree since the *TerL* gene was absent in the genome of *S. maltophilia* phage DLP4).

Genomic network analysis of phage A1432 was performed using the Prokaryotic Viral RefSeq211 Merged database (last updated in June 2022) in vConTACT (v.2.0) (Bin Jang et al., 2019) and the database of the top 20 best hit viruses. Duplicate and unassociated sequences are removed. Based on the number of shared protein clusters (PCs) between genomes, Markov cluster (MCL) and clustering with overlapping neighborhood expansion (ClusterONE) (v1.0) (Nepusz et al., 2012) were used to group the closely related genomes into virus clusters (VCs). Visual network map was drawn using Cytoscape software (v3.9.1v) (Shannon et al., 2003). The whole genome phylogenetic analysis was conducted using the VICTOR online server with default parameters (Meier-Kolthoff and Göker, 2017) based on the database of 20 best hit viruses and the 68 *S. maltophilia* phages (including A1432) with publicly-available genome sequences. Duplicate and unassociated sequences were removed. A pairwise comparison of nucleotide sequences was performed using the Genome-BLAST Distance Phylogeny (GBDP) method. Subsequently, a balanced minimum evolution tree with branch support was constructed using the FASTME with SPR postprocessing based on the intergenomic distances derived from equations D0, D4, D6.

The Virus Intergenomic Distance Calculator (VIRIDIC) heatmap was analyzed using a dataset consistent of viruses with the VICTOR analysis. VIRIDIC was utilized to calculate the pairwise average nucleotide identity (ANI) between the genome of A1432, its closely related phages, the 68 *S. maltophilia* phages. Then a clustering report based on ANI was formed (Moraru et al., 2020).

The whole genome sequence of phage A1432 was compared with that of similar phages using Mauve software (Darling et al., 2004). The phages were further compared in more detail at the gene level using Easyfig software (Sullivan et al., 2011). The default parameters were used.

### 2.12 Statistical analysis

All experiments were performed independently in triplicate. The data are expressed as the mean value and the standard deviation (SD). One/two-way ANOVA analysis of variance using GraphPad Prism. A *p*-value of less than 0.05 was considered statistically significant.

## 3 Results

### 3.1 Phylogeny and AST analyses of host bacterium YCR3A-1

The 16S rRNA gene analysis revealed that strain YCR3A-1 had the highest similarity of 99.72% and coverage of 99% to *S. maltophilia* IH128R2A01 (MN829927). The phylogenetic tree constructed using the ML method (Supplementary Figure S1) showed that YCR3A-1 and the fifteen *Stenotrophomonas* spp. used for the host range test located on the same evolutionary branch as *S. maltophilia*, confirming its identification as *S. maltophilia.* The 16S rRNA sequence was submitted to the NCBI under the accession number OQ983545.

The AST assay demonstrated that YCR3A-1 was resistant to imipenem, cefuroxim and ampicillin, but sensitive to levofloxacin, minocycline, gentamicin and sulfamethoxazole.

### 3.2 Morphology and host range of phage A1432

**Figure 1:**
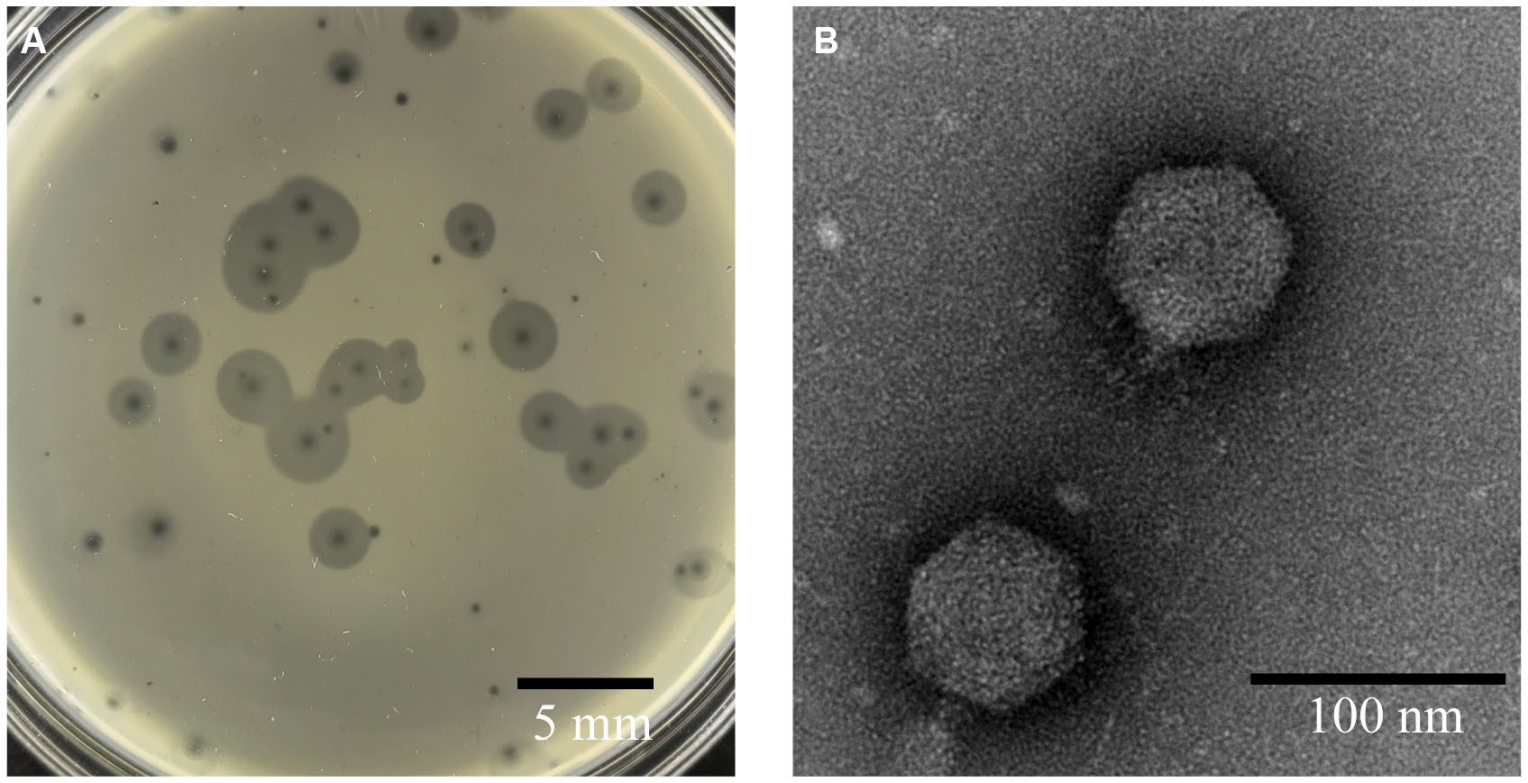
Morphology of phage A1432. **(A)** Phage plaques formed on the lawn of isolate YCR3A-1. **(B)** Transmission electron micrograph.

Phage A1432, infecting *S. maltophilia* YCR3A-1, was isolated from karst cave sediment. This phage could form two types round and transparent plaques on LB double-layer agar plates. One type of plaque had no halo with mean diameter of 1 mm. The other type of plaque has a translucent halo that expands over time, while the size of the lysis zone remained constant (Figure 1). This plaque polymorphism was consistently observed in purification and subsequent experiments. TEM revealed that phage A1432 has an icosahedral head (70.40 ± 1.46 nm) and short tail (14.87 ± 1.84 nm) morphology.

Fifteen strains of *S. maltophilis* isolated from clinical samples and karst caves were used to evaluate phage A1432 host range. The results showed that phage A1432 could only lyse YCR3A-1 and produce clear phage spots, but not the other 15 tested strains. This suggests that A1432 has a very narrow host range.

### 3.3 Optimal MOI and one-step growth curve

**Figure 2:**
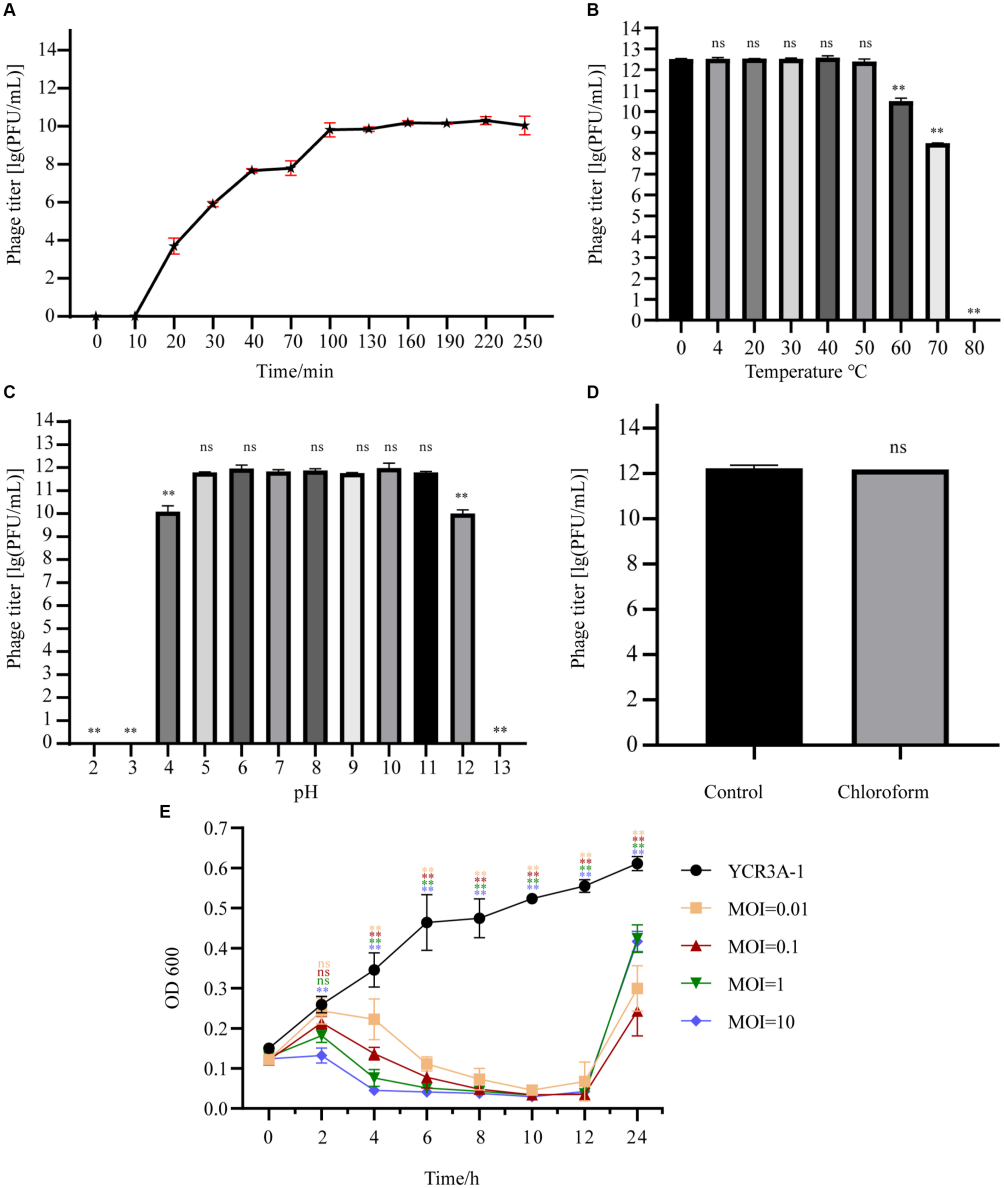
Biological characteristics of phage A1432. **(A)** One-step growth curve of phage A1432. **(B)** Temperature stability. **(C)** pH stability. **(D)** chloroform stability. **(E)** *In vitro* bacteriolytic activity of phage A1432 on host YCR3A-1. All assays were performed in triplicate, the error bars indicate the standard deviation (SD) of three replicates. One-way ANOVA and Dunnett’s *post hoc* test were used. **p < 0.01, ns: no significant difference (*p* > 0.05).

At a MOI 0.1, the titer of A1432 peaked at 1.80 × 10^12^ PFU/mL (Supplementary Table S2), indicating that the optimal MOI of phage A1432 is 0.1. The one-step growth curve (Figure 2A) of phage A1432 was determined at the optimal MOI. There was no significant change in the phage titer within the initial 10 min (*p* > 0.05), after which the titer gradually increased (*p* < 0.01), reaching a plateau at 100 min. The final count of released phage particles was 9.2 × 10^9^ PFU/mL. These results indicate that the phage had a latent period of approximately 10 min, a burst time of 90 min, an average burst size of 43.2 PFU/cell.

### 3.4 Stability of phage A1432

The stability of phage A1432 under various environmental conditions was characterized. The activity of A1432 remained almost constant when the temperature was between 0°C and 50°C (*p* > 0.05). However, the titer of the phage significantly decreased at 60°C (*p* < 0.01) and it was completely inactive at 80°C (Figure 2B). The pH stability test showed that the titer of phage A1432 was relatively stable within a pH range of 5 to 11 (*p* > 0.05). However, when the pH dropped below 4 or rose above 12, the phage titer gradually decreased (*p* < 0.01). Moreover, the phage was almost inactive at pH 3 and 13 (Figure 2C). These results indicated that phage A1432 can withstand a wide pH range, from weakly acidic to alkaline conditions. After treatment with chloroform, phage A1432 remained largely stable compare to the control group (Figure 2D; *p* > 0.05). This suggests that phage A1432 is resistant to chloroform.

### 3.5 *In vitro* lytic capability of phage A1432 against host YCR3A-1

The *in vitro* bacteriolytic activity of phage A1432 against *S. maltophilia* YCR3A-1 at various MOIs was assessed (Figure 2E). The OD600 of YCR3A-1 culture consistently increased within 24 h. However, when phage A1432 was added, the OD600 of the YCR3A-1 culture decreased (*p* < 0.01) in an MOI-dependent manner after 2 h of co-culture. After 12 h of co-culture, the OD600 of the culture increased significantly (*p* < 0.01). The killing curve suggested that phage A1432 was capable to inhibit the growth of host bacteria to varying degrees at different MOI.

### 3.6 Genomic features and functional ORF annotation of phage A1432

**Figure 3:**
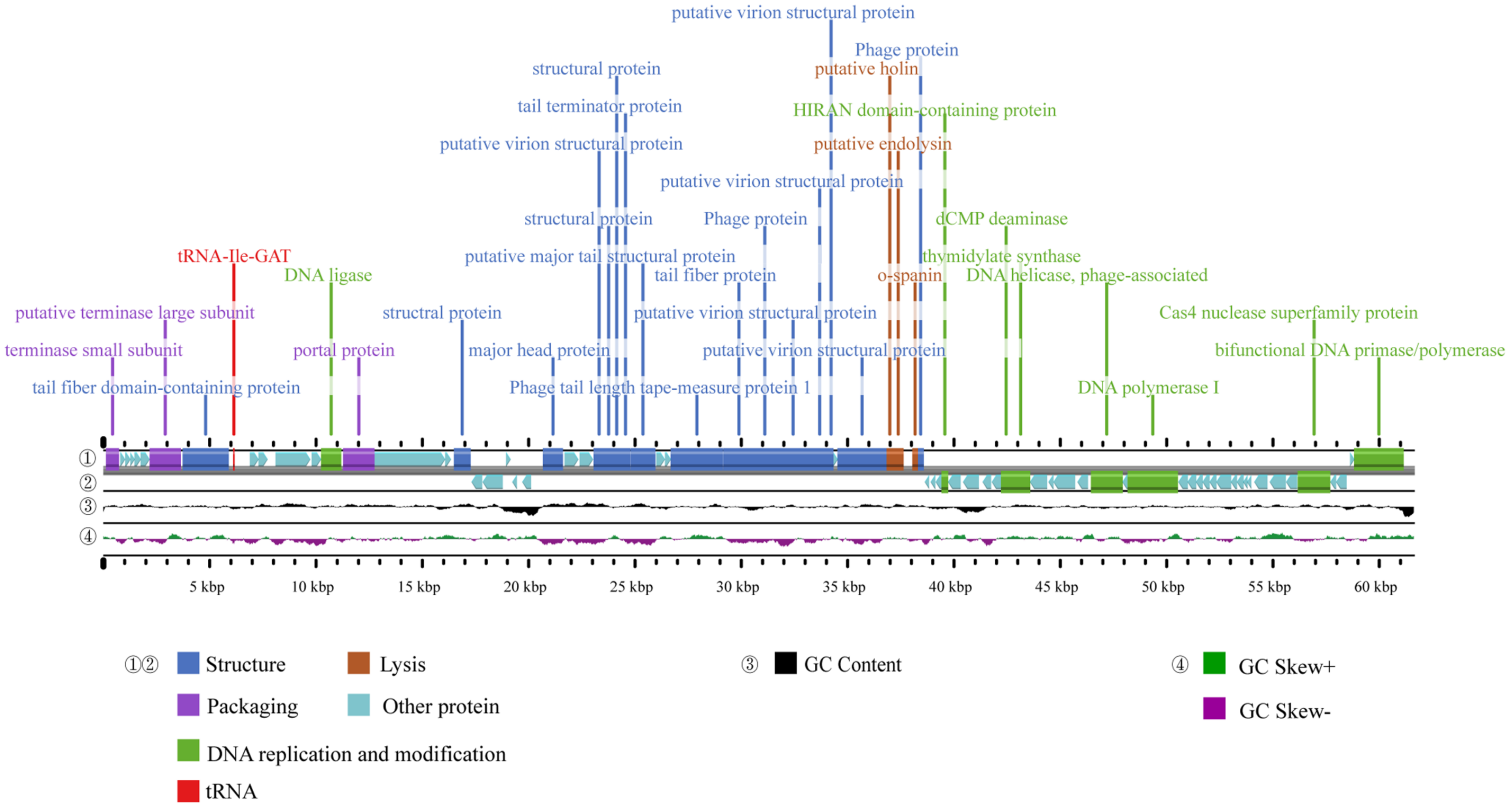
Genome map of phage A1432. ① and ② display the coding sequences (CDS) on the positive and negative strands, with different colors denoting distinct gene functions. ③ illustrates the GC content, while the innermost ④ represents the GC-Skew value.

The complete genome sequence of phage A1432 has been deposited in Genebank under the accession number NC_073027.1. The genome sequencing revealed that phage A1432 is linear double-stranded DNA (dsDNA) with a size of 61,660 base pairs (bp) and a GC content of 61.92%. The RAST annotation showed that the genome contains 79 ORFs. Among these ORFs, 42 are on the positive strand and 37 are on the negative strand. Majority of ORFs (65/79) present ATG start codons, while the remaining (14/79) start with GTG. The total length of the ORFs is 57,667 bp, with an average length of 699.41 bp, accounting for 90.74% of the whole genome. The online tRNAscan-SE tool analysis indicated the presence of a sequence encoding isoleucine-tRNA (72 bp) in the genome (Figure 3). In addition, no virulence, antibiotic resistance, or integrase genes were annotated in the A1432 genome.

The ORFs were functionally annotated by searching a non-redundant protein database using BLASTp. Out of the 79 ORFs, 31 were annotated as proteins with known putative functions, 43 were annotated as hypothetical proteins, 5 unique ORFs were annotated as hypothetical proteins with unknown functions. Based on the putative functions of the 31 ORFs encoding proteins, the genome sequence of phage A1432 can be classified into four modules including structural proteins (16 ORFs), DNA replication and modification (8 ORFs), DNA packaging (3 ORFs), lysis (3 ORFs). All annotated genes were represented in the genome-wide map (Figure 3), the specific grouping of the 79 ORFs was shown in Supplementary Table S3.

### 3.7 Phylogenetic analysis of phage A1432

The whole genome sequence of phage A1432 showed the highest similarity with the *Xanthomonas* phage Xoo-sp2, a member of the *Bradleyvirina*e subfamily, with a nucleotide identity of 81.46% and a query coverage of 37%, indicating that the genome sequence of this phage is relatively novel.

**Figure 4:**
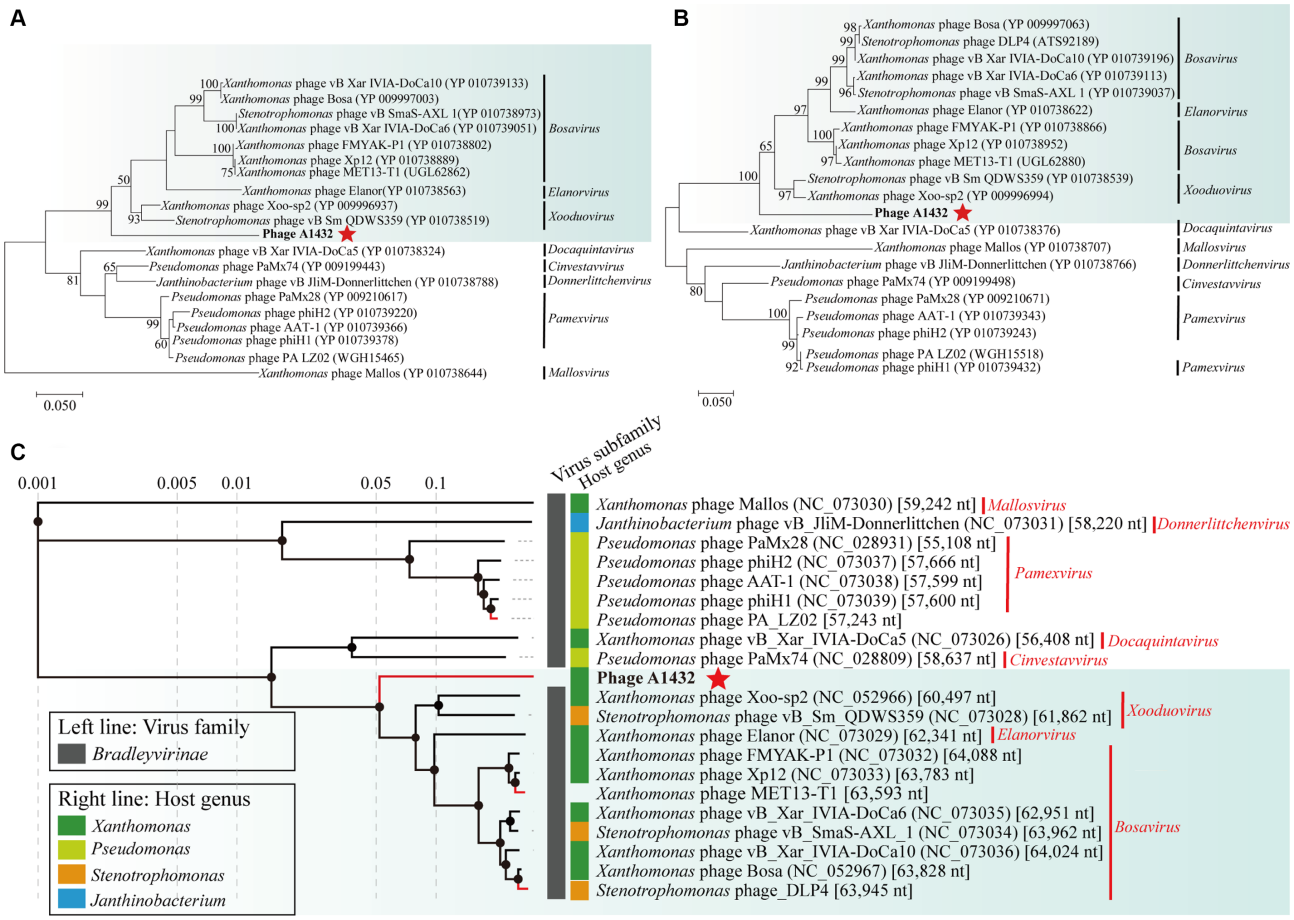
Phylogenetic analysis of phage A1432. Phylogenetic trees of the terminase large subunit **(A)** and DNA polymerase I **(B)**. The phylogenetic trees were constructed based on the amino acid sequences by MEGA 7.0 software. The evolutionary history was inferred by using the Maximum Likelihood method based on the JTT matrix-based model. All parameters are default except the bootstrap value is 1,000. **(C)** Proteomic tree of phage A1432 and its related phages by Viptree. The top 20 hit viruses in Genbank were manually added (red branches) to the Viptree database (last updated in November 17, 2023). Duplicate and unassociated sequences are removed. The classification status of viruses is derived from the ICTV (May 2024). Phage A1432 labeled with a red star. Branch lengths are shown on a logarithmic scale from the root of the tree. The inner nodes of the tree are shown as filled circles, each links to a genomic alignment of the sequences included in its subtree.

The phylogenetic trees of TerL (ORF7) (Figure 4A), DNA polymerase I (ORF61) (Figure 4B) and proteomics (Figure 4C) showed that phage A1432 clustered with 20 phages of subfamily *Bradleyvirinae*, including genus *Xooduovirus*, *Bosavirus*, *Elanorvirus*, *Docaquintavirus*, *Pamexvirus*, *Cinvestavvirus*, *Mallosvirus*, *Donnerlittchenvirus.* Phage A1432 was evolutionarily related to *Xanthomonas* phage Xoo-sp2 (NC_052966) and *Stenotrophomonas* phage vB_Sm_QDWS359 (NC_073028) of the *Xooduovirus* genus, but formed a separate branch.

**Figure 5:**
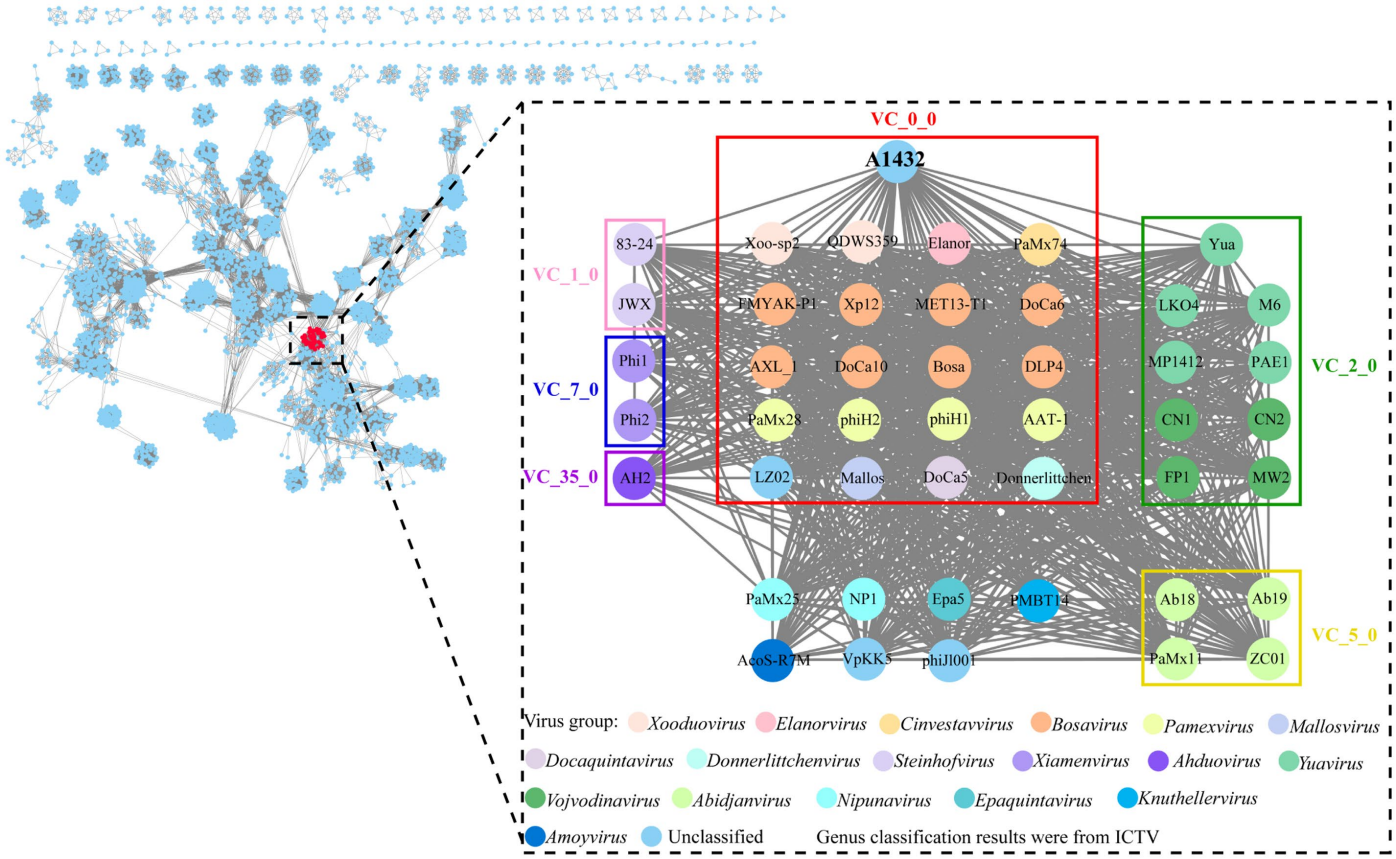
Genomic network map. Genomic network analysis was performed using vConTACT2, MCL, ClusterONE and Cytoscape. The reference database is Prokaryotic Viral RefSeq211-Merged (updated in June 2022), the top 20 similar sequences in Genbank were manually added. Duplicate and unassociated sequences are removed. Viruses are represented as circles (nodes) connected with each other (edges) based on a significant number of shared protein clusters. Circles in various colors indicate viruses of different genera and the classification status of viruses is derived from the ICTV (May 2024). Gray lines connect nodes in the network. Edges represents the strength between two genomes measured by significance score. Clusters of viruses are shown as boxes of different colors: VC_0_0 (red), VC_1_0 (pink), VC_2_0 (green), VC_5_0 (yellow), VC_7_0 (blue), VC_35_0 (purple).

Genomic network analysis based on shared protein showed phage A1432 was found to be associated with 45 phages (dotted box) (Figure 5). Of these, 38 phages were classified into 6 virus clusters (VC_0_0, VC_1_0, VC_2_0, VC_5_0, VC_7_0, VC_35_0), 7 phages could not be classified into any virus cluster. Phage A1432 was classified into a virus cluster (VC_0_0) with the top 20 closely relative viruses.

### 3.8 Phylogenetic analysis of phage A1432 and *Stenotrophomonas maltophilia* phages

**Figure 6:**
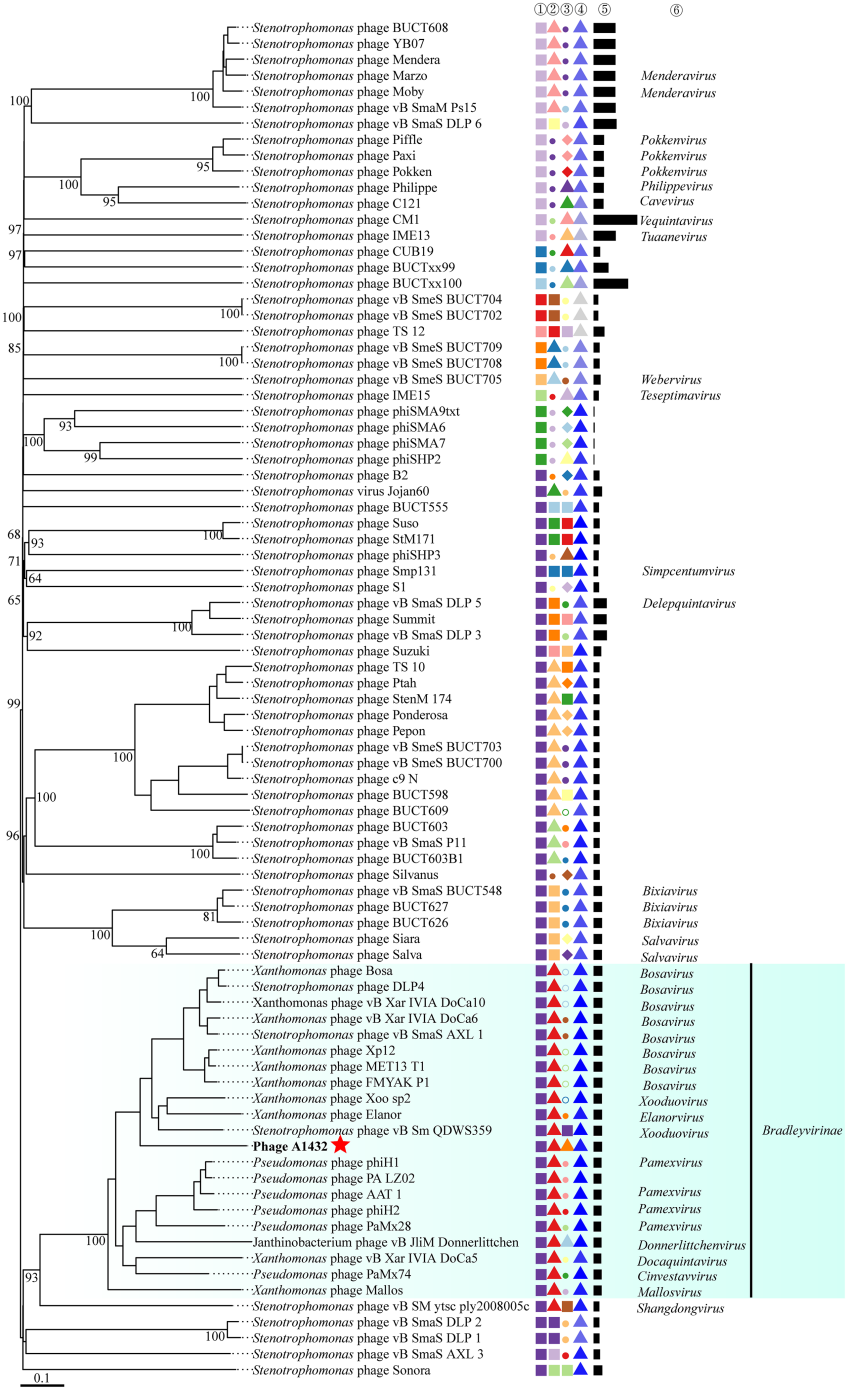
Phylogenetic analysis of the genome sequences by VICTOR analysis. The analysis was performed on the genome sequences of 85 viruses, included the 20 best hit phages whose genome sequences closely related with phage A1432 by BLASTn analysis, 68 *S. maltophilia* phages (including A1432) with publicly-available genome sequences. Duplicate and unassociated sequences are removed. All the genome sequences of the phages were downloaded from NCBI database. The intergenomic distances were inferred using formula D6 with 53% support rate. ①②③④⑤ are derived from VICTOR analysis results. ①②③ represent the clustering information of the phages at the family, genus and species rank, different shapes and colors indicate different family/genus/species. ④ represent the G + C% content of the phages (35.38%/light gray - 67.41%/dark blue). ⑤ represent the genome length of the phages (Min: 5,819 bp; Max: 319,518 bp). ⑥ represent the genus name of the phage classified according to ICTV (February 2024), viruses without genus names indicate that they have not classified in ICTV.

VICTOR was used to perform phylogenetic analysis of the whole genomes of 85 phages, including those closely related to phage A1432 and 68 *S. maltophilia* phages (including A1432). The GBDP tree inferred based on formulas D0, D4, D6 shows average support rates of 49, 10, 53%, respectively. Therefore, formula D6 with the highest supported rate was selected for analysis. The 85 phages were grouped 64 clusters at the species rank, 31 at the genus rank, 10 at the family rank (Figure 6). Phage A1432 and the 20 subfamily *Bradleyvirinae* phages (according to the ICTV classification, accessed on May 6, 2024) clustered together. Phage A1432 is most closely related to *S. maltophilia* phage vB_Sm_QDWS359 of the *Xooduovirus* genus.

**Figure 7:**
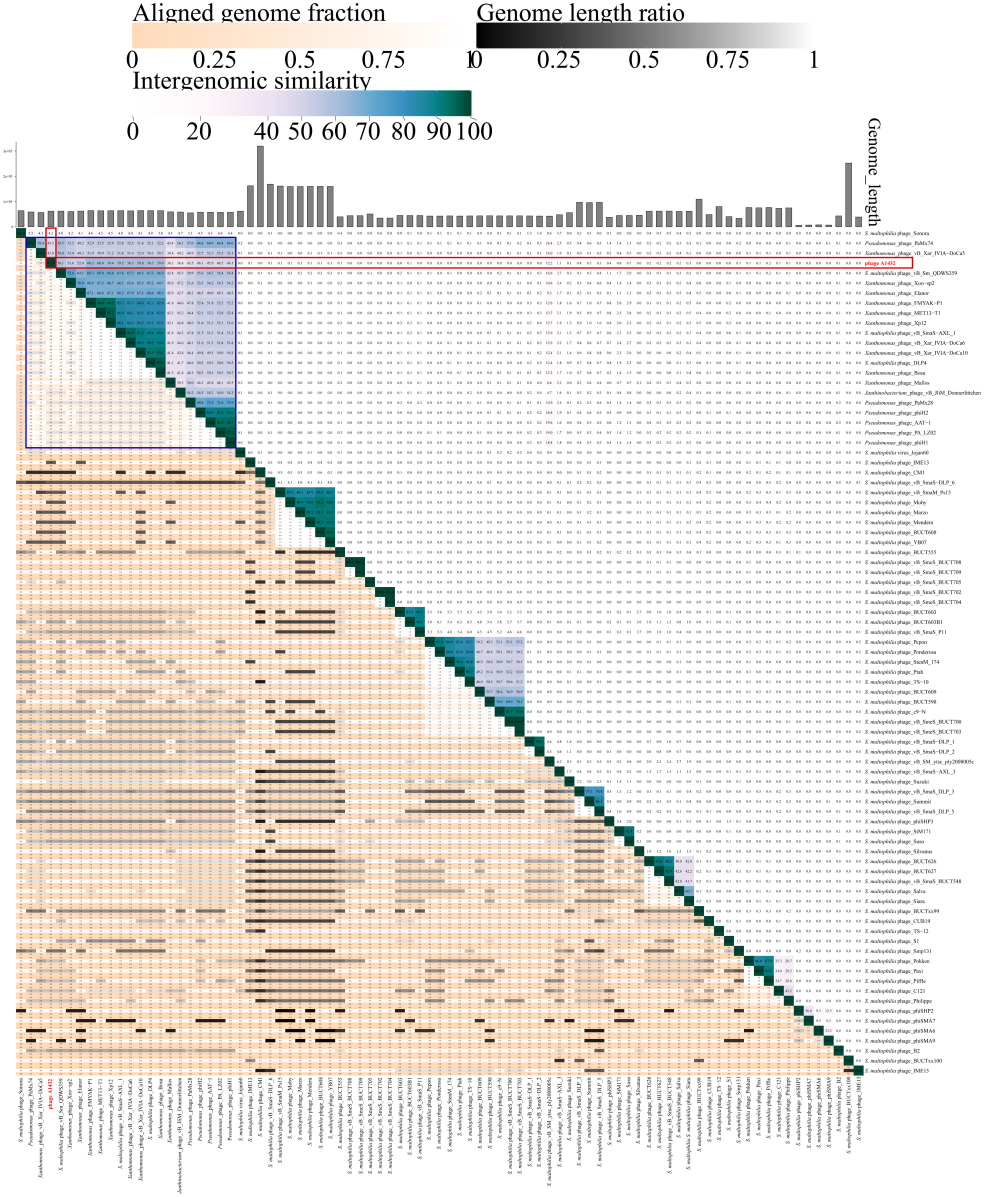
VIRIDIC heatmap of phage A1432. Totally, 85 viruses included the 20 best hit phages whose genome sequences closely related with phage A1432 by BLASTn analysis, 68 *S. maltophilia* phages (including A1432) with publicly-available genome sequences. Duplicate and unassociated sequences are removed. The average nucleotide identity (ANI) of the pairwise intergenomic distances were calculated by VIRIDIC. The red box indicates the ANI value of A1432 compared to the 85 phages. The blue box indicates the ANI value of A1432 compared to the 20 best hit phages.

The VIRIDIC analysis heatmap using a dataset consistent with the VICTOR analysis showed that the 85 phages were grouped into 78 clusters at the species rank and 51 clusters at the genus rank. Phage A1432 formed a separate cluster at the genus rank (Supplementary Table S4), the ANI between phage A1432 and the phages ranged from 0 to 60.5%, with the highest ANI of 60.5% between phage A1432 and *Xanthomonas* phage FMYAK-P1 (NC_073032) of genus *Bosavirus* (Figure 7).

In addition, the VICTOR analysis of 68 *S. maltophilia* phages revealed that there is evidently a high degree of genetic diversity. In addition, the clustering of the 68 *S. maltophilia* phages was relatively scattered, including 10 clusters at the family rank, 31 clusters at the genus rank, 54 clusters at the species rank (Figure 6). These *S. maltophilia* phages genomes have a wide range of sizes, ranging from 5,819 bp to 319,518 bp, with many different sizes within this range. The average GC% content of the *S. maltophilia* phages (57.12%) is similar to that of the host *S. maltophilia* (66.14%) (Fang et al., 2023), although there is substantial variation in GC% ranging from 35.38% (phage TS-12) to 67.41% (phage vB_Sm_QDWS359 and phage Suso) (Figure 6). The VIRIDIC cluster showed that 68 *S. maltophilia* phages were grouped in 44 clusters at genus rank and 64 clusters at species rank (Supplementary Table S4). The ANI between phage A1432 and the other *S. maltophilia* phages ranged from 0 to 59.5%, with the highest ANI of 59.5% between phage A1432 and phage vB_SmaS-AXL_1 (Figure 7).

### 3.9 Comparative genomic analysis

**Figure 8:**
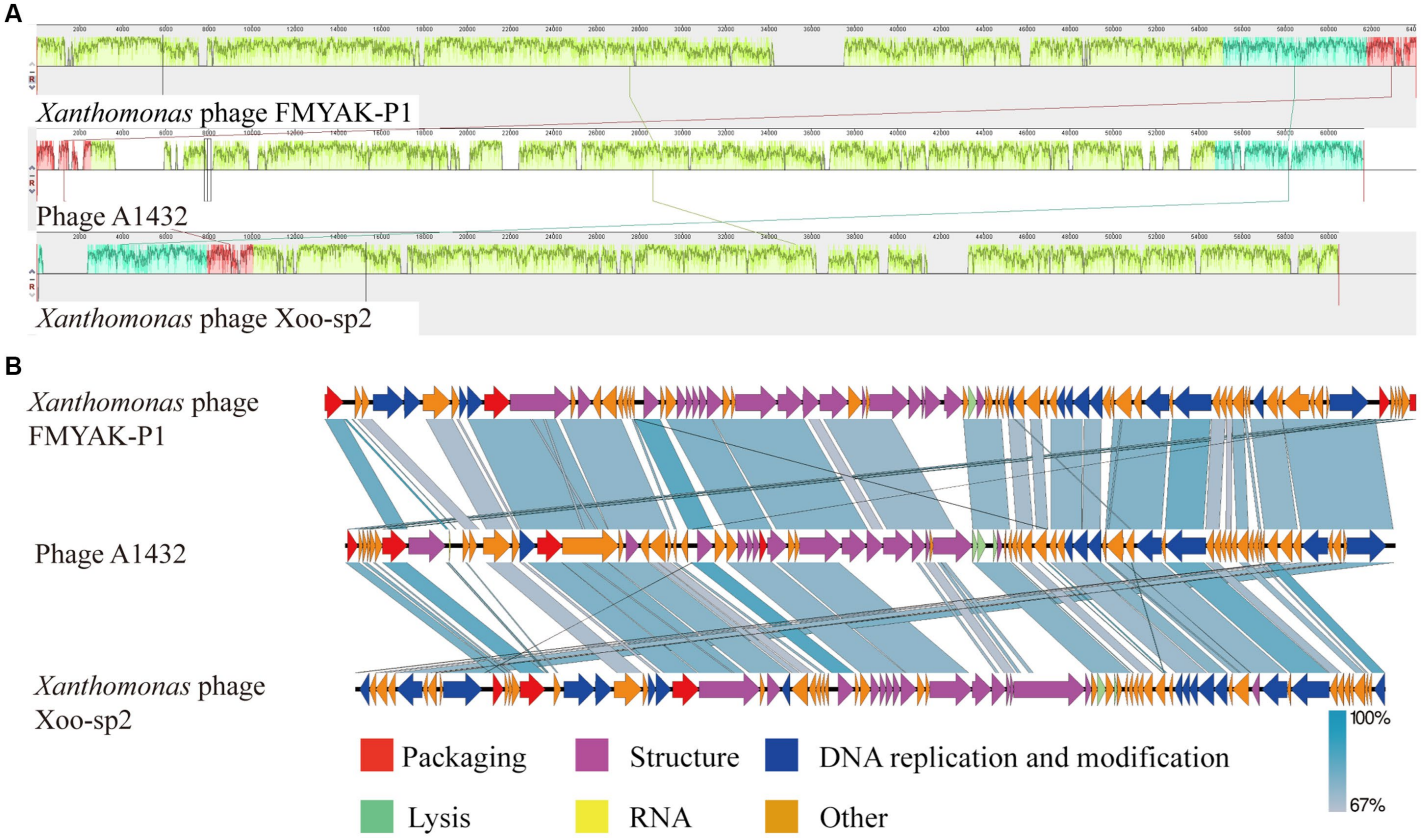
Genome comparison between A1432 and *Xanthomonas* phage Xoo-sp2 and FMYAK-P1. **(A)** Local blocks of colinearity between phages were analyzed using Mauve. The three colinear blocks between the three phages, indicated by different colors. The genome of phage A1432 was rearranged compared to the other two phages. **(B)** Comparison of gene levels of phages using Easyfig software. Arrows indicate predicted ORFs and are shown in different colors depending on the predicted function.

For further comparative genomic analysis, *Xanthomonas* phage Xoo-sp2 and FMYAK-P1 were selected, which showed the highest similarity to phage A1432, according to BlASTn and VIRIDIC results, respectively. Analysis using Mauve software revealed that each of the three phages had three local collinear blocks arranged in different orders (Figure 8A). Further analysis using Easyfig software showed that phage A1432 had an almost identical gene arrangement order with phage Xoo-sp2 and FMYAK-P1, with gene similarity ranging from 67 to 100% (Figure 8B). As a whole, these three phages share similar genes in DNA replication and regulation, structural proteins, DNA packaging, lysis, demonstrating that they may share similar evolutionary traits. In addition, phage A1432 contained a tRNA in its genome, which was absent from the other two phages.

## 4 Discussion

*S. maltophilia* is considered an opportunity pathogen, phage as is a promising alternative therapy for the effective treatment of diseases caused by multi-drug resistant bacteria. In this study, a novel phage A1432 infecting *S. maltophilia* was isolated from a karst cave. The biological characteristics and phylogenetic analyses suggest that phage A1432 may be a novel genus within the subfamily *Bradleyvirinae*, family *Mesyanzhinovviridae*.

Clear plaques and *in vitro* inhibition indicate that A1432 is a lytic phage against *S. maltophilia*. Phage A1432 infection with the host can produce two types of plaques on LB double-layer agar plates: clear plaques and clear plaques with halo rings (Figure 1). Similar plaque polymorphisms have been also observed in other phages previously reported, such as the *S. maltophilia* phage IME13 (Fan et al., 2012) and phage AXL1 (McCutcheon et al., 2022), as well the *Hafnia* phage Ca (Pan et al., 2021). Halo rings had been observed in a variety of phages including *Pseudomonas putida*, *Klebsiella pneumoniae*, *Vibrio alginolyticus* (Cornelissen et al., 2011; Sasikala and Srinivasan, 2016; Majkowska-Skrobek et al., 2018). Halo rings has been used as an indicator of depolymerisation of phage-associated exopolysaccharides (Cornelissen et al., 2011).

Phage A1432 demonstrated a strong host specificity, lysing only YCR3A-1 and not the other 15 strains of *S. maltophilia* isolated from clinical samples and karst caves. Phage cocktail using various phage combinations can successfully minimize the evolution of bacterial resistance and host phage resistance, greater phage functional diversity in the cocktail can make the phage more effective (Guerrero-Bustamante et al., 2021). Phage A1432 has an optimal MOI of 0.1. Compared with the virulent phage Xoo-sp2 which have a latent period of 3 h and burst size of 350 PFU/cell, phage A1432 had a lower burst size (43.2 PFU/cell), but a very short latent period (10 min). The shorter latent period is an advantage when phages are used as therapeutic agents (Bull and Gill, 2014). The results indicate that phage A1432 is a virulent phage then can potentially expand the bactericidal spectrum of phage cocktails (Chan and Abedon, 2012) and expected to be a promising candidate for phage therapy of *S. maltophilia*.

Changes in temperature and pH have a significant effect on the survival of phages. Phage A1432 demonstrated activity stabilization within a temperature range of 0–50°C and pH range of 5–11 (Figures 2B,C), indicating a wide tolerance to various temperature and pH gradients. The high stability of phage A1432 at room temperature and above is a significant advantage for its large-scale production and long-term storage and transportation. A chloroform test revealed that A1432 may be a lipid-free phage, which means chloroform cannot be selected as a preferred disinfectant for A1432. *In vitro* bacterial inhibitory activity assay showed that the phage could completely inhibit the host growth for the initial 12 h under all MOI conditions (Figure 2E). The increase of absorbance in late of infection may be due to the resistance developed by some host cells, or cell fragmentation.

Of the 79 predicted ORFs, 31 were annotated as known functional genes and were distributed across four modules (Figure 3). Eight of these functional genes were associated with DNA replication and modification proteins. These include DNA ligase (ORF13), DNA helicase (ORF59), DNA polymerase (ORF61), primase (ORF79), HIRAN domain-containing protein (ORF48), deoxycytidine monophosphate deaminase (ORF53), thymidylate synthase (ORF54), Cas4 nuclease superfamily protein (75). DNA ligase, encoded by ORF13, aids in DNA replication, repair and recombination by catalyzing the ligation reactions of dsDNA (Shi et al., 2018; Xiang et al., 2020). ORF59 encodes a DNA helicase protein that unravels the double-helical structure of DNA, facilitating its replication. The proteins encoded by ORF61 are predicted to have DNA polymerase activities, which can use parental DNA as a template to catalyze the polymerization of substrate dNTP molecules to form progeny DNA. ORF79 has dual-function activity encoding primase and polymerase, while ORF48 encodes a HIRAN domain-containing protein that has been shown to block replication forks and recognize DNA damage (Iyer et al., 2006). ORF53 and ORF54 encode deoxycytidine monophosphate deaminase (DCD) and thymidylate synthase (TS), respectively, which are involved in DNA synthesis and transcriptional regulation and the thymidylate synthesis pathway. DCD catalyzes the deamination of deoxycytidine monophosphate (dCMP) to produce deoxyuridine monophosphate (dUMP) (Niu et al., 2017) and TS catalyzes the reductive methylation of dUMP to form deoxythymidine monophosphate (dTMP) (Liu et al., 2020). ORF75 encodes a protein of the Cas4 nuclease superfamily, which has 5′-3′ exonuclease activity that generates recombinogenic ends for spacer acquisition (Hooton and Connerton, 2014).

Phage A1432 encodes 16 structure-related genes, accounting for 10.25% of the total genome. ORF23 encodes the major head protein used to package phage nucleic acids and assist viral infection. It has a high amino acid identity (91.94%) with *Stenotrophomonas* phage vB_SmaS-AXL_1. ORF8, ORF29, ORF30, ORF33, ORF34 encode phage tail and tail-related proteins. The tail fiber domain-containing protein (ORF8) is thought to be involved in phage assembly or phage penetration into the outer membrane of the host cell following phage infection and has a significant impact on the host range of the phage (Nobrega et al., 2018). ORF29 plays an essential role in phage tail assembly (Pell et al., 2009). ORF30 encodes a major tail structural protein containing the Phage_tube_2 structural domain, possibly the phage tail tube protein, which polymerizes into a hexameric winding tape measure protein (ORF33) that collectively determines the length of the phage tail as well as facilitates the transfer of DNA to the cytoplasm during infection (Arnaud et al., 2017). The tail fiber protein encoded by ORF34 may be involved in phage-specific recognition of host bacterial surface receptors (Han et al., 2022b). ORF35 and ORF44 encode phage proteins, ORF17, ORF27 and ORF28 encode structural proteins, ORF26, ORF36, ORF37, ORF38 and ORF40 encode putative virion structural proteins.

Virus packaging is a complex process involving the portal proteins, the terminase large subunit and the small subunit. ORF1 encodes the small subunit of the terminase, which is a DNA-binding protein (Roy et al., 2012). ORF7 encodes the large subunit of the terminase, which has ATP-binding, precursor-binding and DNA-splitting activities. ORF14, a portal protein, sits mostly on bottom of capsid and connects to tail to form a channel for phage to inject its own DNA into the host (Sun et al., 2012).

In the lysis module, ORF41 (holin), ORF42 (endolysin) and ORF43 (o-spanin) make up the lysis system of phage A1432. ORF42 encodes endolysin which had 79.46% homology with the protein of *Xanthomonas* phage FMYAK-P1, ORF43 encodes o-spanin protein had 86.34% similarity with the protein of *S. maltophilia* phage vB_Sm_QDWS359 (GenBank: NC_073028.1). Endolysin is speculated to degrade the peptidoglycan layer of the cell wall, holin forms a pore in the bacterial inner membrane, the o-spanin protein catalyzes fusion of the inner and outer membranes and might be involve in extramembrane lysis (Young, 2013). No integrase genes were predicted and annotated in the genome of phage A1432, suggesting that the phage is lytic but not lysogenic.

Indeed, there is no accepted universal method for phage classification considering the advantages and limitations of different classification tools (Barylski et al., 2020). Nucleotide sequence-based approaches capture small differences between closely related genomes, such as silent mutations. Whole genome sequences-based methods (VICTOR, BLAST) are annotation-independent and mitigate the effects of horizontal gene transfer by averaging the signal across the whole genome length. High-throughput network and cluster analyses (vConTACT and VipTree) calculate arbitrary distance matrices from local similarities (Barylski et al., 2020). This may explain the inconsistent discrepancies observed in the results of different methods in this study. For example, phage A1432 showed the most closely relative to the phage from genus *Xooduovirus* (Figures 4, 6) or genus *Bosarvirus* (Figure 7) in the different methods, respectively; VICTOR (Figure 6) and VIRIDIC (Figure 7; Supplementary Table S4) showed inconsistent clustering results at the genus and species ranks; the deviations could be found concerned the phylogenetic evolutionary relationship of the genus of *Elavorvirus, Donnerlittchenvirus*, *Docaquintavirus*, *Cinvestavvirus* (Figures 4, 6).

Therefore, combined tools of maximum-likelihood analysis genome-based annotation-independent method were used for phage A1432 classification. In any case, the phage A1432 genome sequence is relatively unique, the topologies of phylogenetic method and clustering analyses supported the proposed new taxa of phage A1432. The results showed that phage A1432 classified into a virus cluster with the phages from the subfamily *Bradleyvirinae* and is most closely related to the phages of genus *Xooduovirus* and *Bosarvirus* (Figures 4, 6). Moreover, phage A1432 always formed a separate evolutionary branch or cluster at the genus rank (Supplementary Table S4). The ANI values of the genome of A1432 differ by more than 30% from those of the other phages (maximum 60.5%, Figure 7), thus can be classified as a different genus according to the latest classification principles of ICTV (Moraru et al., 2020). Therefore, based on these evidences, we propose that phage A1432 represents a new genus within the subfamily *Bradleyvirinae* (Turner et al., 2021). Recently, phage A1432 has been officially classified by the ICTV and named *Ghuizhouvirus* (ICTV classification, updated May 6, 2024).

Notably, a tRNA is annotated in the genome of phage A1432, which is contradicts the characterization that the species of the family *Mesyanzhinovviridae* do not contain tRNAs (Turner et al., 2023). *Mesyanzhinovviridae* has recently been established and is not well characterized. Many phages encodfe a diverse array of tRNA genes, the content of these genes varies significantly. Some phages carry no tRNA genes, while others possess nearly complete coding sets (Pope et al., 2014; Guerrero-Bustamante and Hatfull, 2024; Wu et al., 2024). The role of these tRNA genes is unclear. However, it has been proposed that they regulate the translation process of structural proteins and specific genes, thereby reducing their dependence on host bacteria and compensating for the inadequacies of host tRNAs required for lytic replication (Kunisawa, 2000; Esposito et al., 2016), or counteract tRNA-degradation-mediated phage defense systems (Van den Berg et al., 2023). Recently, Guerrero-Bustamante and Hatfull showed that phage-encoded tRNA plays a crucial role in establishing lysogenicity in temperate phages (Guerrero-Bustamante and Hatfull, 2024). The roles of tRNA in phage A1432 warrant further investigation. However, whether the absence of tRNA genes can be characterized as a family *Mesyanzhinovviridae* remains to be discussed.

While both phage A1432 and phage Xoo-sp2 are members of the subfamily *Bradleyvirinae* and exhibit a typical icosahedral head structure, their tail morphologies differ. Electron microscopy revealed that phage A1432 has a short tail (Figure 1B), whereas phage Xoo-sp2 has a long tail without contraction (Dong et al., 2018). This discrepancy between morphological traits and genetic analysis has been noted in previous studies as well. For instance, the long-tailed phage HK97 shares no homologous genes or encoded proteins with the morphologically similar phage L5, but it shares numerous identical genes with the short-tailed phage P22 (Lawrence et al., 2002). Historically, phage taxonomy was classified based on morphology. Tailed phages were categorized into three families: *Myoviridae*, *Podoviridae*, *Siphoviridae*, primarily based on their tail morphology observed under electron microscopy: the contractile tail, the long but non-contractile tail, the short but non-contractile tail (Bradley, 1967; Ackermann and Eisenstark, 1974). However, with advancements in genomics and an increasing number of sequenced phage genomes, several independent evaluations have demonstrated that these three morphology-based families are not monophyletic, do not cohere within a monophyletic order, do not accurately reflect a shared evolutionary history (Lawrence et al., 2002; Iranzo et al., 2016; Aiewsakun et al., 2018; Barylski et al., 2020). As a result, the latest classification system by the ICTV has shifted toward a genome-based classification approach. In this system, the traditional families of *Podoviridae*, *Siphoviridae*, *Myoviridae*, as well as the order *Caudovirales*, were abolished and all assigned to the class *Caudoviricetes* (Turner et al., 2023). The results of phage A1432, which exhibits a short-tail morphology but shares high genetic similarity with the long, non-contractile tail phage Xoo-sp2, further support the viewpoint that phage family classification based solely on tail morphology is inappropriate. In any case, our research indicates that phage A1432 can be appropriately classified as a novel genus within the subfamily *Bradleyvirinae,* family *Mesyanzhinovviridae*.

Furthermore, clustering analyses of the nucleotide sequence of 68 *S. maltophilia* phage genomes by using VICTOR and VIRIDIC methods, indicated a considerable genetic diversity and generally low genomic similarity (Figures 6, 7; Supplementary Table S4). The diversity is remarkable considering that all of these phages infect at least one common host (i.e., *S. maltophilia*) that was used to isolate them. One possible explanation for phage diversity is that these phages may have substantially different host ranges, that the GC% reflects their preferred bacterial hosts in their natural environment (Hatfull et al., 2008). In addition, *S. maltophilia* itself exhibits significant genetic diversity among species (Fang et al., 2023). It remains unclear whether the diversity of *S. maltophilia* phages reflects the variety within the host bacterial population. Given the extensive diversity of the *S. maltophilia* phages, the total number of sequenced genomes currently available does not provide substantial insight into the overall organization of the larger phage populations. Future recognition of more phages from multiple environments and hosts will facilitate further exploration of their morphological and genetic diversity and complexity, elucidate the evolutionary mechanisms that give rise to them. This will not only enhance our comprehension of phage phylogenetic evolution but also improve the systematic classification of phages.

## Data availability statement

The datasets presented in this study can be found in online repositories. The names of the repository/repositories and accession number(s) can be found in the article/Supplementary material.

## Author contributions

SL: Writing – review & editing, Writing – original draft, Methodology. MX: Writing – original draft, Methodology. DY: Writing – original draft, Methodology. MY: Writing – original draft, Resources, Methodology. HW: Writing – original draft, Methodology. XL: Writing – original draft, Methodology. CY: Writing – original draft, Methodology. ZF: Writing – original draft, Software. QWu: Writing – original draft, Software. LT: Writing – original draft, Software. WX: Writing – review & editing. QWe: Writing – review & editing, Writing – original draft.

## Footnotes

^1^https://www.ncbi.nlm.nih.gov, accessed 25 December 2023

^2^https://github.com/broadinstitute/pilon

^3^http://rast.nmpdr.org/rast.cgi

^4^http://blast.ncbi.nlm.nih.gov/Blast.cgi

^5^http://lowelab.ucsc.edu/tRNAscan-SE/

^6^http://card.mcmaster.ca/home/

^7^http://www.mgc.ac.cn/VFs/main.htm

^8^https://www.genome.jp/viptree/
