# Supplementary material for: Characterization and genomic analysis of a lytic Stenotrophomonas maltophilia short-tailed phage A1432 revealed a new genus of the family Mesyanzhinovviridae
Source: Front Microbiol. 2024 Jun 27;15:1400700. doi: 10.3389/fmicb.2024.1400700 (PMC11236537; doi:10.3389/fmicb.2024.1400700)
Supplement: Supplementary file 1 [file Table_1.docx]

Supplementary Table 1. Sources of bacterial strains and antimicrobial susceptibility testing of *S. maltophilia* YCR3A-1.

| **Strains** | **Sulfamethoxazole**  **25** μg/**tablet** | **Ampicillin**  **10** μg/**tablet** | **Levofloxacin**  **5** μg/**tablet** | **Cefuroxim**  **30** μg/**tablet** | **Minocycline**  **30** μg/**tablet** | **Imipenem**  **10** μg/**tablet** | **Gentamicin**  **10** μg/**tablet** |  |
| --- | --- | --- | --- | --- | --- | --- | --- | --- |
| *Stenotrophomonas* *maltophilia* 118^a^ | S | R | S | R | S | R | S |  |
| *Stenotrophomonas* *maltophilia* 209^a^ | S | R | S | R | S | R | R |  |
| *Stenotrophomonas* *maltophilia* 53^a^ | S | R | R | R | S | R | R |  |
| *Stenotrophomonas* *maltophilia* 548^a^ | S | R | S | R | S | R | R |  |
| *Stenotrophomonas* *maltophilia* 690^a^ | S | R | S | R | S | R | R |  |
| *Stenotrophomonas* *maltophilia* 824^a^ | S | R | R | R | S | R | R |  |
| *Stenotrophomonas* *maltophilia* 826^a^ | R | R | S | R | S | R | R |  |
| *Stenotrophomonas* *maltophilia* 992^a^ | | S | R | S | R | S | R | S |
| *Stenotrophomonas* *maltophilia* 1785^a^ | | S | R | S | R | S | R | R |
| *Stenotrophomonas* *maltophilia* 1786^a^ | | R | R | S | R | S | R | R |
| YCR3A-1^b^ | | S | R | S | R | S | R | S |
| *Stenotrophomonas* sp. 1-91^b^ | | - | - | - | - | - | - | - |
| *Stenotrophomonas* sp. 1-49^b^ | | - | - | - | - | - | - | - |
| *Stenotrophomonas* sp. 2-24^b^ | | - | - | - | - | - | - | - |
| *Stenotrophomonas* sp. 2-70^b^ | | - | - | - | - | - | - | - |
| *Stenotrophomonas* sp. 5-13^b^ | | - | - | - | - | - | - | - |
| *E. coli* DH5α | | S | S | S | S | S | S | S |

Note: S, susceptible; R, resistant; -, undetected; a: isolated from clinical samples (Han et al., 2021); b: isolated from karst caves.
