# Supplementary material for: Characterization and genomic analysis of a lytic Stenotrophomonas maltophilia short-tailed phage A1432 revealed a new genus of the family Mesyanzhinovviridae
Source: Front Microbiol. 2024 Jun 27;15:1400700. doi: 10.3389/fmicb.2024.1400700 (PMC11236537; doi:10.3389/fmicb.2024.1400700)
Supplement: Supplementary file 2 [file Table_2.docx]

Supplementary Table 2. Optimal MOI of phage A1432.

| Host bacterium (CFU/mL) | Phage (PFU/mL) | MOI | Titer (PFU/mL) |
| --- | --- | --- | --- |
| 10^8^ | 10^10^ | 100 | 9.80×10^11^ |
| 10^8^ | 10^9^ | 10 | 1.18×10^12^ |
| 10^8^ | 10^8^ | 1 | 1.30×10^12^ |
| 10^8^ | 10^7^ | 0.1 | 1.80×10^12^ |
| 10^8^ | 10^6^ | 0.01 | 1.38×10^12^ |
| 10^8^ | 10^5^ | 0.001 | 2.46×10^10^ |
