# Supplementary material for: Characterization and genomic analysis of a lytic Stenotrophomonas maltophilia short-tailed phage A1432 revealed a new genus of the family Mesyanzhinovviridae
Source: Front Microbiol. 2024 Jun 27;15:1400700. doi: 10.3389/fmicb.2024.1400700 (PMC11236537; doi:10.3389/fmicb.2024.1400700)
Supplement: Supplementary file 3 [file Table_3.docx]

Supplementary Table 3. List of annotated proteins encoded by ORFs in the genome of phage A1432.

| **Group** | **Gene product** | **Start**  **Codon** | **Identity (%)** | **Predicted function** | **Accession**  **Number** |
| --- | --- | --- | --- | --- | --- |
| structure | ORF8 | GTG | 58.05 | tail fiber domain-containing protein | [WP_088375287.1](https://www.ncbi.nlm.nih.gov/protein/WP_088375287.1?report=genbank&log$=prottop&blast_rank=1&RID=24MFXGWF013) |
|  | ORF17 | ATG | 86.10 | structural protein | [UGL62849.1](https://www.ncbi.nlm.nih.gov/protein/UGL62849.1?report=genbank&log$=prottop&blast_rank=1&RID=1ZBBWB4S016) |
|  | ORF23 | ATG | 91.94 | major head protein | [UIS24744.1](https://www.ncbi.nlm.nih.gov/protein/UIS24744.1?report=genbank&log$=prottop&blast_rank=1&RID=21UNJU5U013) |
|  | ORF26 | ATG | 79.43 | putative virion structural protein | [YP_009997025.1](https://www.ncbi.nlm.nih.gov/protein/YP_009997025.1?report=genbank&log$=prottop&blast_rank=1&RID=21XN9RAC013) |
|  | ORF27 | ATG | 63.11 | structural protein | [UIS24780.1](https://www.ncbi.nlm.nih.gov/protein/UIS24780.1?report=genbank&log$=prottop&blast_rank=1&RID=21YCYFPK016) |
|  | ORF28 | ATG | 83.08 | structural protein | [UIS24776.1](https://www.ncbi.nlm.nih.gov/protein/UIS24776.1?report=genbank&log$=prottop&blast_rank=1&RID=21YHT3AU016) |
|  | ORF29 | ATG | 79.41 | tail terminator protein | [UGL62837.1](https://www.ncbi.nlm.nih.gov/protein/UGL62837.1?report=genbank&log$=prottop&blast_rank=1&RID=2207C84901R) |
|  | ORF30 | ATG | 82.63 | putative major tail structural protein | [YP_009199465.1](https://www.ncbi.nlm.nih.gov/protein/YP_009199465.1?report=genbank&log$=prottop&blast_rank=1&RID=220D7MET016) |
|  | ORF33 | GTG | 81.48 | phage tail length tape-measure protein 1 | [YP_009997032.1](https://www.ncbi.nlm.nih.gov/protein/YP_009997032.1?report=genbank&log$=prottop&blast_rank=1&RID=2214P82U016) |
|  | ORF34 | ATG | 54.16 | tail fiber protein | YP_009997033.1 |
|  | ORF35 | ATG | 50.45 | Phage protein | [YP_009997034.1](https://www.ncbi.nlm.nih.gov/protein/YP_009997034.1?report=genbank&log$=prottop&blast_rank=1&RID=221FJFN5013) |
|  | ORF36 | GTG | 70.22 | putative virion structural protein | [YP_009199471.1](https://www.ncbi.nlm.nih.gov/protein/YP_009199471.1?report=genbank&log$=prottop&blast_rank=1&RID=221R9UPX016) |
|  | ORF37 | ATG | 71.48 | putative virion structural protein | [YP_009199472.1](https://www.ncbi.nlm.nih.gov/protein/YP_009199472.1?report=genbank&log$=prottop&blast_rank=1&RID=221YUZN8013) |
|  | ORF38 | ATG | 86.84 | putative virion structural protein | [YP_009199473.1](https://www.ncbi.nlm.nih.gov/protein/YP_009199473.1?report=genbank&log$=prottop&blast_rank=1&RID=2222TYNX016) |
|  | ORF40 | GTG | 59.44 | putative virion structural protein | [YP_009199475.1](https://www.ncbi.nlm.nih.gov/protein/YP_009199475.1?report=genbank&log$=prottop&blast_rank=1&RID=222VF1RD013) |
|  | ORF44 | GTG | 72.22 | phage protein | [YP_009997048.1](https://www.ncbi.nlm.nih.gov/protein/YP_009997048.1?report=genbank&log$=prottop&blast_rank=1&RID=239DE1H601R) |
| Lysis | ORF41 | ATG | 85.00 | putative holin | [UIS24783.1](https://www.ncbi.nlm.nih.gov/protein/UIS24783.1?report=genbank&log$=prottop&blast_rank=2&RID=33PKK9Y6013) |
|  | ORF42 | ATG | 80.49 | putative endolysin | [YP_009199477.1](https://www.ncbi.nlm.nih.gov/protein/YP_009199477.1?report=genbank&log$=prottop&blast_rank=1&RID=222ZAA9101R) |
|  | ORF43 | GTG | 81.71 | o-spanin | [UIS24779.1](https://www.ncbi.nlm.nih.gov/protein/UIS24779.1?report=genbank&log$=prottop&blast_rank=1&RID=2238PA7K013) |
| packaging | ORF1 | ATG | 82.44 | terminase small subunit | YP_009996933.1 |
|  | ORF7 | ATG | 84.90 | putative terminase large subunit | [UGL62799.1](https://www.ncbi.nlm.nih.gov/protein/UGL62799.1?report=genbank&log$=prottop&blast_rank=1&RID=24MB0XCJ013) |
|  | ORF14 | ATG | 76.43 | portal protein | [UIS24732.1](https://www.ncbi.nlm.nih.gov/protein/UIS24732.1?report=genbank&log$=prottop&blast_rank=1&RID=1ZAX5UG801R) |
| DNA replication  and modification | ORF13 | ATG | 52.49 | DNA ligase | UIS24745.1 |
|  | ORF48 | ATG | 66.00 | HIRAN domain-containing protein | ATS92247.1 |
|  | ORF53 | ATG | 77.63 | dCMP deaminase | [UIS24764.1](https://www.ncbi.nlm.nih.gov/protein/UIS24764.1?report=genbank&log$=prottop&blast_rank=1&RID=24CAKM1T016) |
|  | ORF54 | GTG | 74.29 | thymidylate synthase | [ATS92206.1](https://www.ncbi.nlm.nih.gov/protein/ATS92206.1?report=genbank&log$=prottop&blast_rank=1&RID=24CERC8501R) |
|  | ORF59 | GTG | 76.31 | DNA helicase, phage-associated | [YP_009997062.1](https://www.ncbi.nlm.nih.gov/protein/YP_009997062.1?report=genbank&log$=prottop&blast_rank=1&RID=24E5NJ8Y013) |
|  | ORF61 | ATG | 82.85 | DNA polymerase I | [YP_009996994.1](https://www.ncbi.nlm.nih.gov/protein/YP_009996994.1?report=genbank&log$=prottop&blast_rank=1&RID=24EBF35R013) |
|  | ORF75 | ATG | 77.69 | Cas4 nuclease superfamily protein | [ATS92198.1](https://www.ncbi.nlm.nih.gov/protein/ATS92198.1?report=genbank&log$=prottop&blast_rank=1&RID=24JBW8KE01R) |
|  | ORF79 | GTG | 84.11 | bifunctional DNA primase/polymerase | [YP_009997077.1](https://www.ncbi.nlm.nih.gov/protein/YP_009997077.1?report=genbank&log$=prottop&blast_rank=1&RID=24K6G5AW01R) |
| other protein | ORF2 | ATG | 57.14 | hypothetical protein AXL1_02 | [UIS24797.1](https://www.ncbi.nlm.nih.gov/protein/UIS24797.1?report=genbank&log$=prottop&blast_rank=1&RID=24KREFJ2016) |
|  | ORF3 | ATG | 78.75 | hypothetical protein JTY55_gp11 | YP_009996934.1 |
|  | ORF4 | ATG | 90.6 | hypothetical protein JTY56_gp79 | [YP_009997081.1](https://www.ncbi.nlm.nih.gov/protein/YP_009997081.1?report=genbank&log$=prottop&blast_rank=1&RID=24KYPGHM016) |
|  | ORF5 | ATG | 54.10 | hypothetical protein JTY55_gp12 | [YP_009996935.1](https://www.ncbi.nlm.nih.gov/protein/YP_009996935.1?report=genbank&log$=prottop&blast_rank=1&RID=24M3M9J8016) |
|  | ORF6 | ATG | 47.45 | hypothetical protein 38_00068 | [QIQ65871.1](https://www.ncbi.nlm.nih.gov/protein/QIQ65871.1?report=genbank&log$=prottop&blast_rank=1&RID=24M6MX0W013) |
|  | ORF9 | ATG | 52.24 | hypothetical protein | [QNN97160.1](https://www.ncbi.nlm.nih.gov/protein/QNN97160.1?report=genbank&log$=prottop&blast_rank=1&RID=24MNC26E016) |
|  | ORF10 | ATG | 55.07 | hypothetical protein | [QNN97161.1](https://www.ncbi.nlm.nih.gov/protein/QNN97161.1?report=genbank&log$=prottop&blast_rank=1&RID=24MSZCKJ013) |
|  | ORF11 | ATG | 58.32 | hypothetical protein | [UGL62720.1](https://www.ncbi.nlm.nih.gov/protein/UGL62720.1?report=genbank&log$=prottop&blast_rank=1&RID=1ZA70ZS7016) |
|  | ORF12 | ATG | 44.44 | hypothetical protein JTY56_gp07 | [YP_009997009.1](https://www.ncbi.nlm.nih.gov/protein/YP_009997009.1?report=genbank&log$=prottop&blast_rank=1&RID=1ZADVXKZ016) |
|  | ORF15 | ATG | 73.58 | hypothetical protein | [QNN97169.1](https://www.ncbi.nlm.nih.gov/protein/QNN97169.1?report=genbank&log$=prottop&blast_rank=1&RID=1ZB118HA016) |
|  | ORF16 | ATG | 83.12 | hypothetical protein JTY55_gp24 | [YP_009996947.1](https://www.ncbi.nlm.nih.gov/protein/YP_009996947.1?report=genbank&log$=prottop&blast_rank=1&RID=1ZB8PWM901R) |
|  | ORF18 | ATG | 75.50 | uncharacterized protein COG3236 | YP_009997016.1 |
|  | ORF19 | GTG | 62.28 | hypothetical protein AXL1_20 | [UIS24746.1](https://www.ncbi.nlm.nih.gov/protein/UIS24746.1?report=genbank&log$=prottop&blast_rank=1&RID=1ZBMT990013) |
|  | ORF21 | ATG | 79.41 | hypothetical protein JTY55_gp30 | [YP_009996953.1](https://www.ncbi.nlm.nih.gov/protein/YP_009996953.1?report=genbank&log$=prottop&blast_rank=1&RID=21TFK6X901R) |
|  | ORF22 | ATG | 29.63 | hypothetical protein JTY56_gp17 | [YP_009997019.1](https://www.ncbi.nlm.nih.gov/protein/YP_009997019.1?report=genbank&log$=prottop&blast_rank=1&RID=21U371GZ013) |
|  | ORF24 | ATG | 46.03 | hypothetical protein CPT_Sonora_008 | [QYW02111.1](https://www.ncbi.nlm.nih.gov/protein/QYW02111.1?report=genbank&log$=prottop&blast_rank=1&RID=21X3AD76016) |
|  | ORF25 | ATG | 56.16 | hypothetical protein | [UGL62841.1](https://www.ncbi.nlm.nih.gov/protein/UGL62841.1?report=genbank&log$=prottop&blast_rank=1&RID=21X7KS99016) |
|  | ORF72 | ATG | 84.31 | hypothetical DUF2815 containing protein | [UIS24754.1](https://www.ncbi.nlm.nih.gov/protein/UIS24754.1?report=genbank&log$=prottop&blast_rank=1&RID=24HNV04V01R) |
|  | ORF31 | ATG | 73.95 | hypothetical protein JTY56_gp28 | [YP_009997030.1](https://www.ncbi.nlm.nih.gov/protein/YP_009997030.1?report=genbank&log$=prottop&blast_rank=1&RID=220MJKSC013) |
|  | ORF32 | ATG | 75.00 | hypothetical protein AVV52_gp24 | [YP_009196277.1](https://www.ncbi.nlm.nih.gov/protein/YP_009196277.1?report=genbank&log$=prottop&blast_rank=1&RID=220UU4BD01R) |
|  | ORF39 | ATG | 66.15 | hypothetical protein | [QNN97194.1](https://www.ncbi.nlm.nih.gov/protein/QNN97194.1?report=genbank&log$=prottop&blast_rank=1&RID=2227TANC016) |
|  | ORF45 | ATG | 57.58 | hypothetical protein JTY55_gp54 | YP_009996977.1 |
|  | ORF46 | ATG | 61.9 | hypothetical protein JTY55_gp55 | [YP_009996978.1](https://www.ncbi.nlm.nih.gov/protein/YP_009996978.1?report=genbank&log$=prottop&blast_rank=1&RID=239P62A4016) |
|  | ORF47 | ATG | 32.04 | hypothetical protein JTY55_gp56 | [YP_009996979.1](https://www.ncbi.nlm.nih.gov/protein/YP_009996979.1?report=genbank&log$=prottop&blast_rank=1&RID=239TT3EX013) |
|  | ORF49 | ATG | 64.55 | hypothetical protein | [QNN97208.1](https://www.ncbi.nlm.nih.gov/protein/QNN97208.1?report=genbank&log$=prottop&blast_rank=1&RID=23A7X07R01R) |
|  | ORF50 | ATG | 55.14 | hypothetical protein | [QNN97209.1](https://www.ncbi.nlm.nih.gov/protein/QNN97209.1?report=genbank&log$=prottop&blast_rank=1&RID=23AJUKAV01R) |
|  | ORF51 | ATG | 74.59 | hypothetical protein | ATS92232.1 |
|  | ORF52 | ATG | 51.23 | hypothetical protein JTY56_gp54 | [YP_009997056.1](https://www.ncbi.nlm.nih.gov/protein/YP_009997056.1?report=genbank&log$=prottop&blast_rank=1&RID=24A44NFR013) |
|  | ORF55 | GTG | 73.47 | HAD superfamily hydrolase | [YP_009996988.1](https://www.ncbi.nlm.nih.gov/protein/YP_009996988.1?report=genbank&log$=prottop&blast_rank=1&RID=24CM175K01R) |
|  | ORF56 | ATG | 50.00 | hypothetical protein JTY55_gp66 | YP_009996989.1 |
|  | ORF57 | ATG | 78.57 | hypothetical protein AXL1_67 | UIS24738.1 |
|  | ORF58 | ATG | 80.00 | hypothetical protein AVV49_gp56 | [YP_009199495.1](https://www.ncbi.nlm.nih.gov/protein/YP_009199495.1?report=genbank&log$=prottop&blast_rank=1&RID=24E28NT9016) |
|  | ORF60 | ATG | 53.42 | hypothetical protein WJ96_04950 | [KVP97922.1](https://www.ncbi.nlm.nih.gov/protein/KVP97922.1?report=genbank&log$=prottop&blast_rank=1&RID=24E8GJ3U016) |
|  | ORF62 | GTG | 65.07 | hypothetical protein | UGL62780.1 |
|  | ORF63 | ATG | 72.66 | hypothetical protein JTY56_gp63 | [YP_009997065.1](https://www.ncbi.nlm.nih.gov/protein/YP_009997065.1?report=genbank&log$=prottop&blast_rank=1&RID=24G297FR013) |
|  | ORF65 | ATG | 62.26 | hypothetical protein | [UGL62877.1](https://www.ncbi.nlm.nih.gov/protein/UGL62877.1?report=genbank&log$=prottop&blast_rank=1&RID=24GGTFPH013) |
|  | ORF67 | ATG | 71.18 | hypothetical protein | [UGL62783.1](https://www.ncbi.nlm.nih.gov/protein/UGL62783.1?report=genbank&log$=prottop&blast_rank=1&RID=24GWDGX5013) |
|  | ORF69 | ATG | 38.61 | hypothetical protein HYP11_gp087 | [YP_009876991.1](https://www.ncbi.nlm.nih.gov/protein/YP_009876991.1?report=genbank&log$=prottop&blast_rank=1&RID=24H081ES016) |
|  | ORF70 | ATG | 64.91 | hypothetical protein | [ATS92265.1](https://www.ncbi.nlm.nih.gov/protein/ATS92265.1?report=genbank&log$=prottop&blast_rank=1&RID=24H6H4YC016) |
|  | ORF73 | ATG | 53.42 | hypothetical protein AXL1_78 | UIS24750.1 |
|  | ORF74 | ATG | 70.53 | hypothetical protein JTY56_gp70 | [YP_009997072.1](https://www.ncbi.nlm.nih.gov/protein/YP_009997072.1?report=genbank&log$=prottop&blast_rank=1&RID=24J6NV15013) |
|  | ORF76 | ATG | 51.19 | hypothetical protein | [UGL62870.1](https://www.ncbi.nlm.nih.gov/protein/UGL62870.1?report=genbank&log$=prottop&blast_rank=1&RID=24JN3SZZ01R) |
|  | ORF77 | GTG | 43.96 | hypothetical protein | [UGL62869.1](https://www.ncbi.nlm.nih.gov/protein/UGL62869.1?report=genbank&log$=prottop&blast_rank=1&RID=24JV597D01R) |
|  | ORF78 | ATG | 60.00 | hypothetical protein | [UGL62868.1](https://www.ncbi.nlm.nih.gov/protein/UGL62868.1?report=genbank&log$=prottop&blast_rank=1&RID=24K0RVH8016) |
|  | ORF20 | ATG | no hit | - | - |
|  | ORF64 | ATG | no hit | - | - |
|  | ORF66 | ATG | no hit | - | - |
|  | ORF68 | GTG | no hit | - | - |
|  | ORF71 | ATG | no hit | - | - |
|  | tRNA |  |  | tRNA-Ile-GAT |  |
