# Supplementary material for: Characterization and genomic analysis of a lytic Stenotrophomonas maltophilia short-tailed phage A1432 revealed a new genus of the family Mesyanzhinovviridae
Source: Front Microbiol. 2024 Jun 27;15:1400700. doi: 10.3389/fmicb.2024.1400700 (PMC11236537; doi:10.3389/fmicb.2024.1400700)
Supplement: Supplementary file 4 [file Table_4.docx]

Supplementary Table 4. Clustering results of phage A1432 and its phylogenetically related phages as well as 68 *S. maltophilia* phages using VIRIDIC.

| Phage genome | Genus cluster | Species cluster |
| --- | --- | --- |
| *Janthinobacterium*phage vB_JliM_Donnerlittchen | G 1 | S 1 |
| **phage_A1432** | **G 2** | **S 2** |
| *Stenotrophomonas* phage B2 | G 3 | S 3 |
| *Stenotrophomonas* phage BUCT555 | G 4 | S 4 |
| *Stenotrophomonas* phage BUCT598 | G 5 | S 5 |
| *Stenotrophomonas* phage BUCT603 | G 6 | S 6 |
| *Stenotrophomonas* phage BUCT603B1 | G 6 | S 7 |
| *Stenotrophomonas* phage vB_SmaS_P11 | G 6 | S 55 |
| *Stenotrophomonas* phage BUCT608 | G 7 | S 8 |
| *Stenotrophomonas* phage YB07 | G 7 | S 8 |
| *Stenotrophomonas* phage Marzo | G 7 | S 21 |
| *Stenotrophomonas* phage Mendera | G 7 | S 22 |
| *Stenotrophomonas* phage Moby | G 7 | S 23 |
| *Stenotrophomonas* phage vB_SmaM_Ps15 | G 7 | S 51 |
| *Stenotrophomonas* phage BUCT609 | G 8 | S 9 |
| *Stenotrophomonas* phage BUCT626 | G 9 | S 10 |
| *Stenotrophomonas* phage BUCT627 | G 9 | S 11 |
| *Stenotrophomonas* phage vB_SmaS_BUCT548 | G 9 | S 52 |
| *Stenotrophomonas* phage BUCTxx100 | G 10 | S 12 |
| *Stenotrophomonas* phage BUCTxx99 | G 11 | S 13 |
| *Stenotrophomonas* phage C121 | G 12 | S 14 |
| *Stenotrophomonas* phage c9-N | G 13 | S 15 |
| *Stenotrophomonas* phage vB_SmeS_BUCT700 | G 13 | S 61 |
| *Stenotrophomonas* phage vB_SmeS_BUCT703 | G 13 | S 61 |
| *Stenotrophomonas* phage CM1 | G 14 | S 16 |
| *Stenotrophomonas* phage CUB19 | G 15 | S 17 |
| *Stenotrophomonas* phage DLP4 | G 16 | S 18 |
| *Xanthomonas* phage Bosa | G 16 | S 18 |
| *Stenotrophomonas* phage vB_SmaS-AXL_1 | G 16 | S 56 |
| *Xanthomonas* phage FMYAK-P1 | G 16 | S 72 |
| *Xanthomonas* phage MET13-T1 | G 16 | S 74 |
| *Xanthomonas* phage Xp12 | G 16 | S 74 |
| *Xanthomonas* phage vB_Xar_IVIA-DoCa10 | G 16 | S 75 |
| *Xanthomonas* phage vB_Xar_IVIA-DoCa6 | G 16 | S 77 |
| *Stenotrophomonas* phage IME13 | G 17 | S 19 |
| *Stenotrophomonas* phage IME15 | G 18 | S 20 |
| *Stenotrophomonas* phage Paxi | G 19 | S 24 |
| *Stenotrophomonas* phage Piffle | G 19 | S 32 |
| *Stenotrophomonas* phage Pokken | G 19 | S 33 |
| *Stenotrophomonas* phage Pepon | G 20 | S 25 |
| *Stenotrophomonas* phage Ponderosa | G 20 | S 34 |
| *Stenotrophomonas* phage Ptah | G 20 | S 35 |
| *Stenotrophomonas* phage StenM_174 | G 20 | S 42 |
| *Stenotrophomonas* phage TS-10 | G 20 | S 47 |
| *Stenotrophomonas* phage Philippe | G 21 | S 26 |
| *Stenotrophomonas* phage phiSHP2 | G 22 | S 27 |
| *Stenotrophomonas* phage phiSHP3 | G 23 | S 28 |
| *Stenotrophomonas* phage phiSMA6 | G 24 | S 29 |
| *Stenotrophomonas* phage phiSMA7 | G 25 | S 30 |
| *Stenotrophomonas* phage phiSMA9 | G 26 | S 31 |
| *Stenotrophomonas* phage S1 | G 27 | S 36 |
| *Stenotrophomonas* phage Salva | G 28 | S 37 |
| *Stenotrophomonas* phage Siara | G 29 | S 38 |
| *Stenotrophomonas* phage Silvanus | G 30 | S 39 |
| *Stenotrophomonas* phage Smp131 | G 31 | S 40 |
| *Stenotrophomonas* phage Sonora | G 32 | S 41 |
| *Stenotrophomonas* phage StM171 | G 33 | S 43 |
| *Stenotrophomonas* phage Suso | G 33 | S 45 |
| *Stenotrophomonas* phage Summit | G 34 | S 44 |
| *Stenotrophomonas* phage vB_SmaS_DLP_3 | G 34 | S 53 |
| *Stenotrophomonas* phage vB_SmaS_DLP_5 | G 34 | S 54 |
| *Stenotrophomonas* phage Suzuki | G 35 | S 46 |
| *Stenotrophomonas* phage TS-12 | G 36 | S 48 |
| *Stenotrophomonas* phage vB_Sm_QDWS359 | G 37 | S 49 |
| *Xanthomonas* phage Xoo-sp2 | G 37 | S 78 |
| *Stenotrophomonas* phage vB_SM_ytsc_ply2008005c | G 38 | S 50 |
| *Stenotrophomonas* phage vB_SmaS-AXL_3 | G 39 | S 57 |
| *Stenotrophomonas* phage vB_SmaS-DLP_1 | G 40 | S 58 |
| *Stenotrophomonas* phage vB_SmaS-DLP_2 | G 40 | S 59 |
| *Stenotrophomonas* phage vB_SmaS-DLP_6 | G 41 | S 60 |
| *Stenotrophomonas* phage vB_SmeS_BUCT702 | G 42 | S 62 |
| *Stenotrophomonas* phage vB_SmeS_BUCT704 | G 42 | S 62 |
| *Stenotrophomonas* phage vB_SmeS_BUCT705 | G 43 | S 63 |
| *Stenotrophomonas* phage vB_SmeS_BUCT708 | G 44 | S 64 |
| *Stenotrophomonas* phage vB_SmeS_BUCT709 | G 44 | S 64 |
| *Pseudomonas* phage AAT-1 | G 45 | S 65 |
| *Pseudomonas* phage PA_LZ02 | G 45 | S 66 |
| *Pseudomonas* phage phiH1 | G 45 | S 66 |
| *Pseudomonas* phage phiH2 | G 45 | S 69 |
| *Pseudomonas* phage PaMx28 | G 46 | S 67 |
| *Pseudomonas* phage PaMx74 | G 47 | S 68 |
| *Stenotrophomonas* virus Jojan60 | G 48 | S 70 |
| *Xanthomonas* phage Elanor | G 49 | S 71 |
| *Xanthomonas* phage Mallos | G 50 | S 73 |
| *Xanthomonas* phage vB_Xar_IVIA-DoCa5 | G 51 | S 76 |
