# Supplementary figures and images for: Characterization and genomic analysis of a lytic Stenotrophomonas maltophilia short-tailed phage A1432 revealed a new genus of the family Mesyanzhinovviridae
Source: Front Microbiol. 2024 Jun 27;15:1400700. doi: 10.3389/fmicb.2024.1400700 (PMC11236537; doi:10.3389/fmicb.2024.1400700)

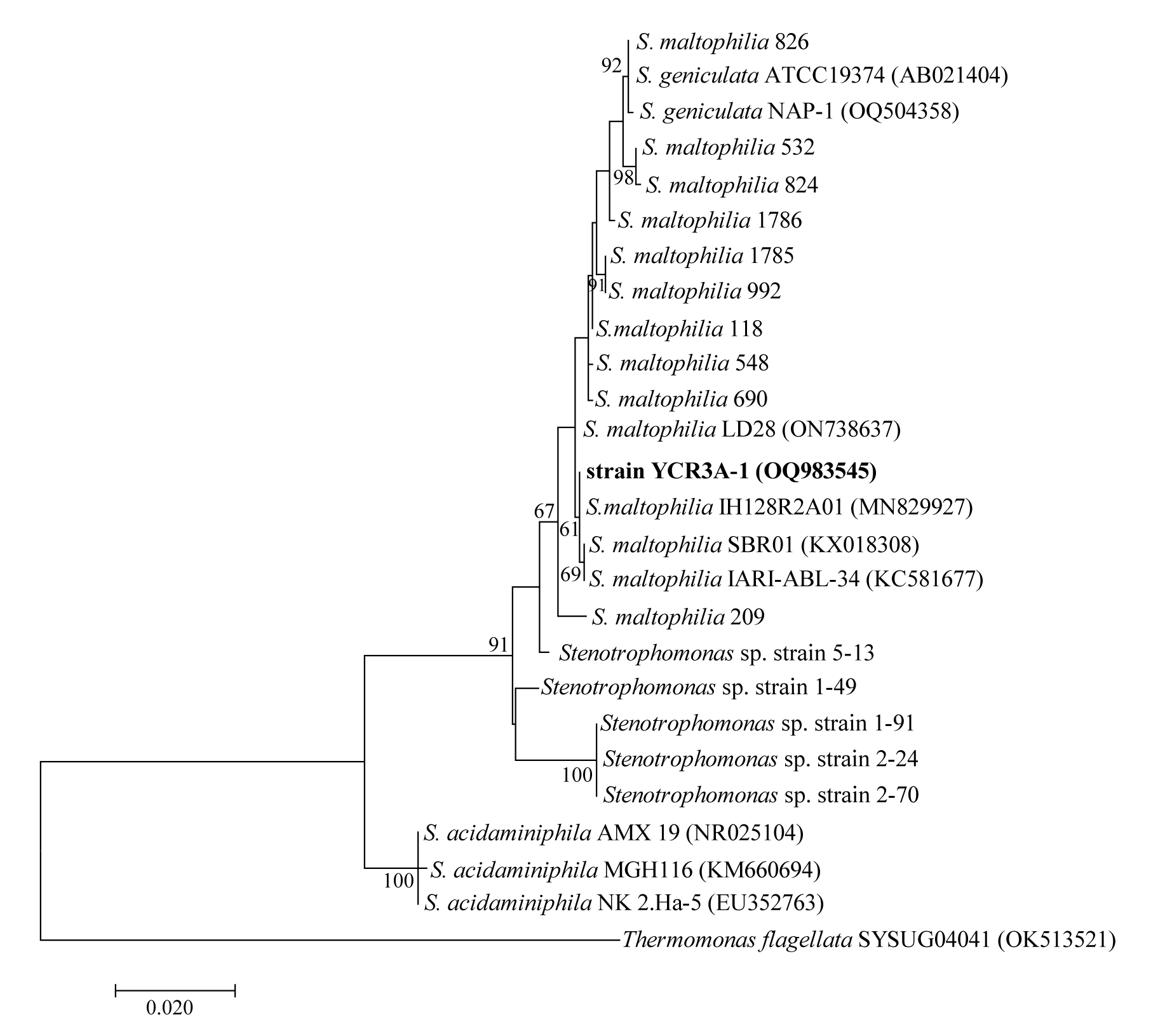

Supplement: Supplementary file 5 [file Image_1.TIF]
